# Unique dynamic profiles of social attention in autistic females

**DOI:** 10.1111/jcpp.13630

**Published:** 2022-05-30

**Authors:** Teresa Del Bianco, Luke Mason, Meng‐Chuan Lai, Eva Loth, Julian Tillmann, Tony Charman, Hannah Hayward, Teresa Gleissl, Jan K. Buitelaar, Declan G.M. Murphy, Simon Baron‐Cohen, Sven Bölte, Mark H. Johnson, Emily J. H. Jones, The EU‐AIMS LEAP Group

**Affiliations:** ^1^ Centre for Brain and Cognitive Development Birkbeck University of London London UK; ^2^ 33561 Centre for Addiction and Mental Health and The Hospital for Sick Children Department of Psychiatry University of Toronto Toronto ON Canada; ^3^ Autism Research Centre Department of Psychiatry University of Cambridge Cambridge UK; ^4^ Department of Psychiatry National Taiwan University Hospital and College of Medicine Taipei Taiwan; ^5^ Institute of Psychiatry, Psychology and Neuroscience King’s College London London UK; ^6^ Department of Cognitive Neuroscience Donders Institute for Brain, Cognition and Behaviour Nijmegen The Netherlands; ^7^ Center of Neurodevelopmental Disorders (KIND) Department of Women’s Health Karolinska Institutet Solna Sweden; ^8^ Department of Psychology University of Cambridge Cambridge UK

**Keywords:** Autism, social attention, eye‐tracking, sex differences, male, female

## Abstract

**Background:**

Social attention affords learning opportunities across development and may contribute to individual differences in developmental trajectories, such as between male and female individuals, and in neurodevelopmental conditions, such as autism.

**Methods:**

Using eye‐tracking, we measured social attention in a large cohort of autistic (*n* = 123) and nonautistic females (*n* = 107), and autistic (*n* = 330) and nonautistic males (*n* = 204), aged 6–30 years. Using mixed Growth Curve Analysis, we modelled sex and diagnostic effects on the temporal dynamics of proportional looking time to three types of social stimuli (lean‐static, naturalistic‐static, and naturalistic‐dynamic) and examined the link between individual differences and dimensional social and nonsocial autistic traits in autistic females and males.

**Results:**

In the lean‐static stimulus, average face‐looking was higher in females than in males of both autistic and nonautistic groups. Differences in the dynamic pattern of face‐looking were seen in autistic vs. nonautistic females, but not males, with face‐looking peaking later in the trial in autistic females. In the naturalistic‐dynamic stimulus, average face‐looking was higher in females than in males of both groups; changes in the dynamic pattern of face looking were seen in autistic vs. nonautistic males, but not in females, with a steeper peak in nonautistic males. Lower average face‐looking was associated with higher observer‐measured autistic characteristics in autistic females, but not in males.

**Conclusions:**

Overall, we found stronger social attention in females to a similar degree in both autistic and nonautistic groups. Nonetheless, the dynamic profiles of social attention differed in different ways in autistic females and males compared to their nonautistic peers, and autistic traits predicted trends of average face‐looking in autistic females. These findings support the role of social attention in the emergence of sex‐related differences in autistic characteristics, suggesting an avenue to phenotypic stratification.

## Introduction

Autism is a neurodevelopmental condition with a prevalence rate of 1 in 89 children (Maenner et al., 2020; Posada de la Paz, 2018) characterised by difficulties with social communication and interaction, restricted, repetitive behaviours and sensory alterations, causing support needs (American Psychiatric Association, 2013). Sex stratifies autism on multiple levels, such as in prevalence rates (which are 3 times more frequent in males than in females; Loomes, Hull, & Mandy, 2017), core symptom domains (Lai & Szatmari, 2020), and brain organisation (e.g., cortical connectivity, Floris et al., 2021, and morphometry, Hammill et al., 2021). Understanding the neurocognitive mechanisms that differ between males and females may highlight mechanisms contributing to differing symptoms and presentation and inform clinical support (Constantino, Charman, & Jones, 2021).

Social attention − dynamic engagement with other people − has been a leading candidate neurocognitive marker of autistic neurodevelopment. Several studies have found that social attention is decreased in autistic people (Frazier et al., 2017) and altered prior to formal clinical diagnosis (Bedford et al., 2016; Chawarska, Macari, Powell, DiNicola, & Shic, 2016). However, interpretation and generalisation have been partly limited by low female representation (Frazier et al., 2017). Recent studies that have included a higher number of female participants found that clinically diagnosed autistic females showed similar average looking time to faces (Harrop et al., 2019), and social vs. nonsocial preferences (Harrop et al., 2018, 2020), as nonautistic females, unlike the reductions in social attention shown in autistic vs. nonautistic males. In these studies, a similar pattern of sex differences in social attention was observed in autism and neurotypical development, and diagnostic group differences were smaller in females than in males. These observations suggest that sex differences in social attention may not be explained by models such as the Extreme Male Brain Hypothesis (Baron‐Cohen, 2002) and the Additive Inherited Liability model (Constantino et al., 2021), which predict that autistic people may not show the same pattern of sex differences as neurotypical people, and that diagnostic group differences may be bigger in females compared to males. Other models suggest that social attention may act as a protective (Chawarska et al., 2016) or moderating factor (Johnson, Charman, Pickles, & Jones, 2021) because it may maintain engagement with others and provide opportunities for learning. However, sex differences in the relation between social attention and dimensional variation in autistic symptoms have not been explored.

Sex differences in social attention might be most sensitively tested by including not only averaged measures of social attention but using methods that capture the temporal dynamics of social interest. Indeed, recent studies (Del Bianco et al., 2021; Hedger & Chakrabarti, 2021) have shown that analytical methods that treat social attention as a time series are sensitive to age‐related and contextual flexibility, aiding the elucidation of the underlying neurocognitive processes. In the present work, we examined the modulation of social attention by sex and diagnosis and its relation to dimensional symptomatology in a large sample of the multi‐site Longitudinal European Autism Project, LEAP (Loth et al., 2017), which included a large sample of female participants of a wide age range. We predicted that, consistent with the model described above, social attention would be greater in females than males in both autistic and nonautistic groups, and that the pattern of diagnostic group differences would differ in males and females, across stimuli that vary in their complexity (Harrop et al., 2019) and ecological validity (Chevallier et al., 2015), that have been, respectively, found to better elicit sex and diagnostic group differences. Furthermore, we predicted that fewer social communication traits in autistic females would be associated with more significant deviance from autistic males, compared to nonautistic females, formalised as a pattern of sex differences exceeding diagnostic group differences.

## Methods

### Participants

The data come from 764 participants (453 autistic and 311 nonautistic), in four countries (the United Kingdom, Germany, The Netherlands, Italy). See Table 1 for demographic characteristics and comparisons by diagnostic group and sex.

**Table 1:** Essential demographic characteristics of the sample, split by diagnostic group and sex. Effect sizes of between sex comparisons (Phi Coefficient for categorical variables, Cohen’s D for continuous variables) are provided for each group separately

Parameter	Females		Phi Coefficients (Categorical) / Cohen’s *D* (Continuous)	Males		Phi Coefficients (Categorical) / Cohen’s *D* (Continuous)
	Autistic	Nonautistic		Autistic	Nonautistic	
N	123	107	–	330	204	0.006
FSIQ < 75	26 (21%)	15 (14%)	0.008	50 (15%)	20 (9%)	0.005
Age Range in Year	6.08 ~ 30.28	6.89 ~ 30.78	–	6.13 ~ 30.60	6.24 ~ 30.98	–
Age Mean in Year (*SD*)	16.77 (6.36)	17.04 (5.92)	0.04	16.69 (5.62)	17.23 (5.96)	0.09
FSIQ Mean (*SD*)	95.88 (20.37)	104.39 (19.94)	0.42	97.17 (19.85)	103.48 (18.3)	0.33
SRS T‐Score Mean (*SD*)	73.73 (12.13)	48.07 (8.94)	2.38	71.52 (11.49)	48.35 (9.78)	2.13
RBS‐R Mean (*SD*)	15.92 (13.53)	2.34 (4.96)	1.30	17.01 (13.92)	3.1 (9.95)	1.11
ADOS SA‐CSS Mean (*SD*)	5.45 (2.53)	–	–	6.42 (2.64)	–	–
ADOS RRB‐CSS Mean (*SD*)	4.1 (2.58)	–	–	5 (2.81)	–	–

Parameter	NonAutistic		Phi Coefficients (Categorical) / Cohen’s *D* (Continuous)	Autistic		Phi Coefficients (Categorical) / Cohen’s *D* (Continuous)
	Female	Male		Female	Male	
*N*	107	204	–	123	330	0.006
FSIQ < 75	15	20	0.003	26	50	0.005
Age Range in Years	6.89 ~ 30.78	6.24 ~ 30.98	–	6.08 ~ 30.28	6.13 ~ 30.60	–
Age Mean in Years (*SD*)	17.04 (5.92)	17.23 (5.96)	0.03	16.77 (6.36)	16.69 (5.62)	0.01
FSIQ Mean (*SD*)	104.39 (19.94)	103.48 (18.3)	0.05	95.88 (20.37)	97.17 (19.85)	0.06
SRS T‐Score Mean (*SD*)	48.07 (8.94)	48.35 (9.78)	0.03	73.73 (12.13)	71.52 (11.49)	0.19
RBS‐R Mean (*SD*)	2.34 (4.96)	3.1 (9.95)	0.09	15.92 (13.53)	17.01 (13.92)	0.08
ADOS SA‐CSS Mean (*SD*)	–	–	–	5.45 (2.53)	6.42 (2.64)	0.37
ADOS RRB‐CSS Mean (*SD*)	–	–	–	4.1 (2.58)	5 (2.81)	0.33

ADOS RRB‐CSS, Autism Diagnostic Observation Schedule Repetitive Restricted Behaviour Calibrated Severity Score; ADOS SA‐CSS, Autism Diagnostic Observation Schedule Social Affect Calibrated Severity Score; FSIQ, Full Scale Intelligence Quotient; *N*, number; RBS‐R, Repetitive Behaviour Scale Revised; SRS, Social Responsiveness Scale.

### Ethical considerations

The study was carried out upon approval of national and local ethics review boards at each study site. Participants or their parents signed a written consent before entering the study.

### Clinical variables

For dimensional symptoms, we used the standardised *T*‐score of the parent‐reported Social Responsiveness Scale 2, SRS‐2 (Constantino & Gruber, 2005), the parent‐reported Repetitive Behaviour Scale‐Revised, RBS‐R (Bourreau, Roux, Gomot, Bonnet‐Brilhault, & Barthélémy, 2009), and Social Affect and Restricted Repetitive Behavior Calibrated Severity Scores (SA‐CSS and RRB‐CSS, respectively) of the Autism Diagnostic Observation Schedule, ADOS (Esler et al., 2015; Lord et al., 2000) Module 2/3/4 (see Charman et al., 2017).

### Eye‐trackers and Software

Sites used a Tobii T120 (3, 383 participants) or TX300 (3,381 participants) eye tracker (Tobii AB, Sweden), at a maximum sampling rate (120 and 300 Hz). The difference in screen size (17” and 23” respectively) was uniformed by presenting the stimuli on a 17” virtual screen with a black border on the TX300. The freedom of head movement was similar at a standard distance from the screen, and slightly bigger for the T120 (44*22 cm) compared to the TX300 (37*17 cm). Stimuli were presented on Apple Macbook Pro (Apple Inc., USA), with TaskEngine (sites.google.com/site/taskenginedoc/). Raw gaze was recorded and processed with Tobii Gaze Analytics SDK 3.0.

### Stimuli

Three sets of stimuli were presented on a virtual screen of 33*18 visual degrees of angle on 1,280 × 1,024 monitors:

- Face pop‐out (FPO): 8 static arrays of one face (balanced by gender), a scrambled face, a car, a bird and a mobile phone for 10 seconds (Gliga, Elsabbagh, Andravizou, & Johnson, 2009; Gui et al., 2020), without audio.
- Static Scenes (SS): 6 naturalistic photographs of adults, children (nine females and four males in foreground) and everyday objects, for 20 s (Del Bianco et al., 2021), without audio.
- Dynamic Video (DV): a 40‐seconds extract from “50 People, One Question Brooklin” (http://fiftypeopleonequestion.com/), presenting street interviews with 14 people (balanced by gender), with relaxing piano music in the background.

### Procedure

The participant sat centrally at 60 cm from the screen. Five‐point calibration was performed up to 3 times before the experimenter could skip the presentation. The presentation − intermixed with other stimuli − proceeded automatically when the participants fixated the interstimulus image, in 4 blocks of 7 min (Face Pop‐Out and Static Scenes: 1st block; Dynamic Video: last block), all including post‐hoc calibration checks, for an overall duration of 28 min.

### Pre‐processing

**Figure 1:**
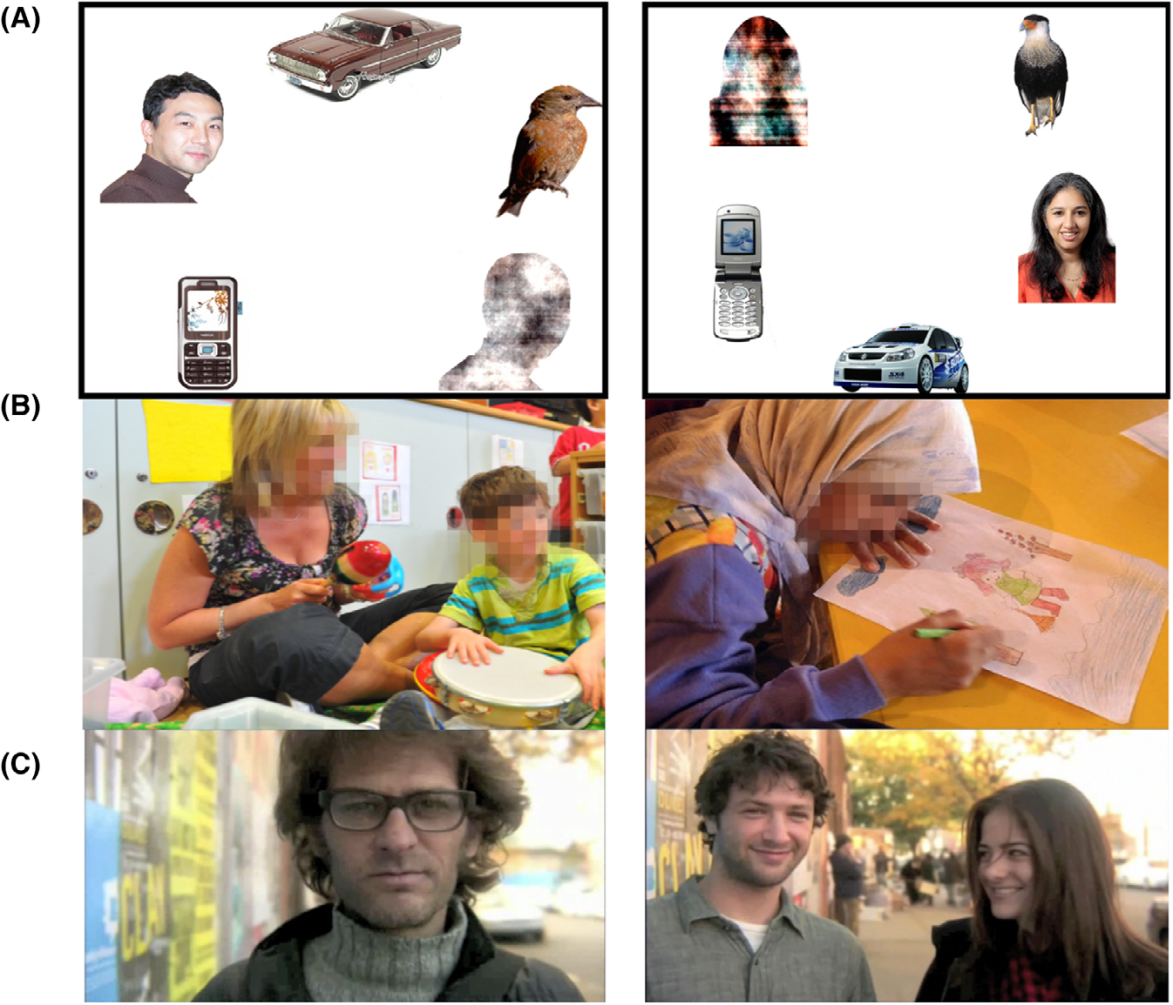
(A) 2 sample stimuli from the eight gendered‐balanced slides of the Face Pop‐Out. (B) two samples of six photographs portraying nine females and four males of the Static Scenes (faces are pixelated for privacy reasons). (C) screenshot of three of 14 individual people (gender‐balanced) interviewed on the street in the Dynamic Video

AOIs were manually drawn on the faces (Figure 1). Each sample was scored according to whether the gaze coordinates fell within the face and aggregated in time bins of half a second, obtaining the Proportional Looking Time (PLT). For the Dynamic Video, we defined 3‐second segments from the onset of a face, excluding scenes <3 s, and cutting exceeding time >3 s. We excluded time bins with missing data (i.e., eyes not detected/out of the screen) >75%.

### Statistical analysis

#### Analytic models

We applied Growth Curve Analysis, GCA (Mirman, Dixon, & Magnuson, 2008) in a series of successive models to assess sex and diagnostic group differences (Lai et al., 2013).

We calculated the orthogonal polynomials of the time of presentation of the stimulus, up to degree 3 of the Face Pop‐Out and the Static Scenes (3rd degree polynomials correspond to 3 changes of focus, or Slope, Quadratic and Cubic Components), and up to degree 2 for the Dynamic Video (2nd degree polynomials correspond to 2 changes of focus, or Slope, Quadratic Components). Additionally, we included random intercepts and slopes by the participant, and random intercepts by trial/scene (Equation 1).

(1) $$y_{i,s}=\;\beta_{0}+\beta_{1}P_{n}x_{i}+\beta_{0i}+\beta_{0s}+\beta_{1i}P_{n}x_{i}+\;\varepsilon_{i}+\;\varepsilon_{s}$$

where $y_{i,s}$ = PLT of the ‐ith participant (i), for the ‐sth stimulus (s), $\beta_{0}$ = fixed intercept, $\beta_{1}$ = fixed slope, $P_{n}x_{i}$ = polynomial function, $\beta_{0i}$, $\beta_{0s}$ = random intercepts, $\beta_{1i}P_{n}x_{i}=$ random polynomials, $\varepsilon_{i}$ = overall variability, $\varepsilon_{s}$ = stimulus variability.

With reference to Lai et al. (2013), we fitted two sex and two diagnostic group differences models through a process of stepwise addition tested with Likelihood Ratio Tests: (a) diagnosis/sex, (b) orthogonal polynomials (interaction), (c) age (covariate/interaction), (d) proportion of missing data (covariate; Appendix S1 Tables FPO S4, S5, S8, S9; SS S12, S13, S16, S17; DV S20, S21, S24, S25), (e) FSIQ (covariate; Appendix S1 section S1.4). For significant effects, we report the Coefficients (Coef.; referenced to the female/nonautistic group, corresponding to unstandardised effect sizes), Bootstrapped 95% Confidence Intervals (between squared brackets) and Standard Errors (*SE*) of the model with the best fit.

### Dimensional variation

We extracted random effects, i.e., individual coefficients, from the sex differences model in the autistic group. Differently from the Coefficients pertaining to the analytical models above, random effects quantify the effect of sex on individual participants. We used them as dependent variables in multiple linear regressions with SRS‐2, RBS‐R, ADOS SA‐CSS and ADOS RRB‐CSS as predictors, in interaction with sex, with stimulus and age as covariates (Equation 2). *T*‐adjustment based on Monte Carlo approximations was applied to pairwise comparisons.

(2) $$y=\;\beta_{0}+\beta_{1}x_{i}j_{i}+j_{i}+k_{1}+k_{2}+\varepsilon$$

where *y* = random effect, $\beta_{0}$ = fixed intercept, $\beta_{1}$ = fixed slope, $x_{i}$ = clinical variable,$j_{i}$ = sex, $k_{1}$ = age,$k_{2}$ = stimulus, ε = variability.

Second, we subtracted the random effects of female participants extracted from the sex differences model from the corresponding random effect extracted from the diagnostic group model, thus obtaining a different score, illustrating how much bigger/smaller the effect of sex is for each female compared to the effect of being autistic. We used it as dependent variables in a multiple linear regression with ADOS SA‐CSS as a predictor, with stimulus and age as covariates (Equation 3).

(3) $$y=\;\beta_{0}+\beta_{1}x_{i}+k_{1}+k_{2}+\varepsilon$$

where *y* = difference score, $\beta_{0}$ = fixed intercept, $\beta_{1}$ = fixed slope, $x_{i}$ = ADOS SA‐CSS, $k_{1}$ = age,$k_{2}$ = stimulus, ε = variability.

## Results

87% of the sample provided usable data (for autistic and nonautistic groups, respectively, final sample sizes 388 and 275 for FPO; 417 and 245 for SS; 388 and 271 for DV). 638 provided valid data for all 3 stimuli (104 autistic females, 89 nonautistic females, 268 autistic males, 177 nonautistic males; missing due to tasks not presented 10.23%; lack of acquisition 90.77%). Based on Monte Carlo Simulations, with the current sample, there is an estimated power of 96.38% to detect previously observed effects (Del Bianco et al., 2021; Harrop et al., 2020); power estimation only starts to drop below 70–80% if assumed effects are 80% smaller than previously found (Confidence Interval = 55.85 – 75.18% for sex differences in the autistic subsample; Confidence Interval = 47.71 – 67.80% for diagnostic group differences in the female subsample). Calibration accuracy (i.e., Euclidean distance from the centroid of all gaze samples to the central interstimulus image) did not vary by group (Coef. = 0.001, *SE* = 0.002, *p*‐value = .44), indicating that there were no significant changes of data quality over the time of the session (see Appendix S1 Table S1).

The % of missing data differed between stimuli, with a higher percentage in SS (23.42%, *SD* = 7.56), and lower in FPO (17.80%, *SD* = 7.79) and DV (10%, *SD* = 14.41), which was accounted for by inclusion as a covariate for each model. Differences between groups reached small effect sizes (<0.4; see Appendix S1, Tables S2 and S3).

After controlling for missing data, FSIQ did not contribute to any of the model fit and was thus excluded (see Appendix S1 section S1.4).

The overall pattern of results is summarised in Table 2. In the following paragraphs, we report the significant coefficients/effects for each model (for the complete list of coefficients, see Appendix S1 Table FPO S6, S7, S10, S11; SS S14, S15, S18, S19; DV S22, S23, S26, S27).

**Table 2:** Descriptive summary of all results

Stimulus	Average Profile (Intercept)		Dynamic Profile (Polynomials)	
	Sex differences models	Diagnostic group differences models	Sex differences models	Diagnostic group differences models
Face Pop‐Out	Males < Females (Nonautistic: Coef. = −0.03, *p*‐value = .01; autistic: Coef. = −0.02, *p*‐value = .01)	Autistic females > nonautistic females (Coef = 0.08, *p*‐value = .03) Age‐dependent decrease in autistic females (Coef. = −0.005, *p*‐value = .01)		Late peak in autistic females (Coef. = −0.15, *p*‐value = .008) Age‐dependent flattening with age in females (Coef. = 0.01, *p*‐value < .001)
	More social attention in females versus males; subtended by different processes in autistic and nonautistic females, with age‐dependent convergence at the average level, but the divergence of the dynamic profile			
Static Scenes	Males < Females (Nonautistic: Coef. = −0.02, *p*‐value = .05; autistic: Coef. = −0.02, *p*‐value = .07)	Age‐dependent increase but not statistically different between groups (Males: Coef. = −0.003, *p*‐value = .07; Females: −0.005, *p*‐value = .06)		
	No robust sex differences			
Dynamic Video	Males < Females (Nonautistic: Coef. = −0.06, *p*‐value < .001; autistic: Coef. = −0.04, *p*‐value = .02)	Nonautistic > autistic (Males: Coef. = −0.07, *p*‐value < .001; Females: −0.09, *p*‐value < .001)	Lower maximum and farther roots in autistic males than females (Coef. = 0.05, *p*‐value = .002)	Lower net increase (Coef. = −0.05, *p*‐value = .007) and maximum (Coef. = 0.05, *p*‐value < .001) in autistic males than in nonautistic males Nonsignificant lower maximum and farther roots in autistic females than nonautistic females (Coef. = 0.04, *p*‐value = .06)
	More social attention in females versus males; subtended by a lower maximum, and lower net increase, in autistic males			

### Face Pop‐Out

#### Sex differences models

##### Average profile

**Figure 2:**
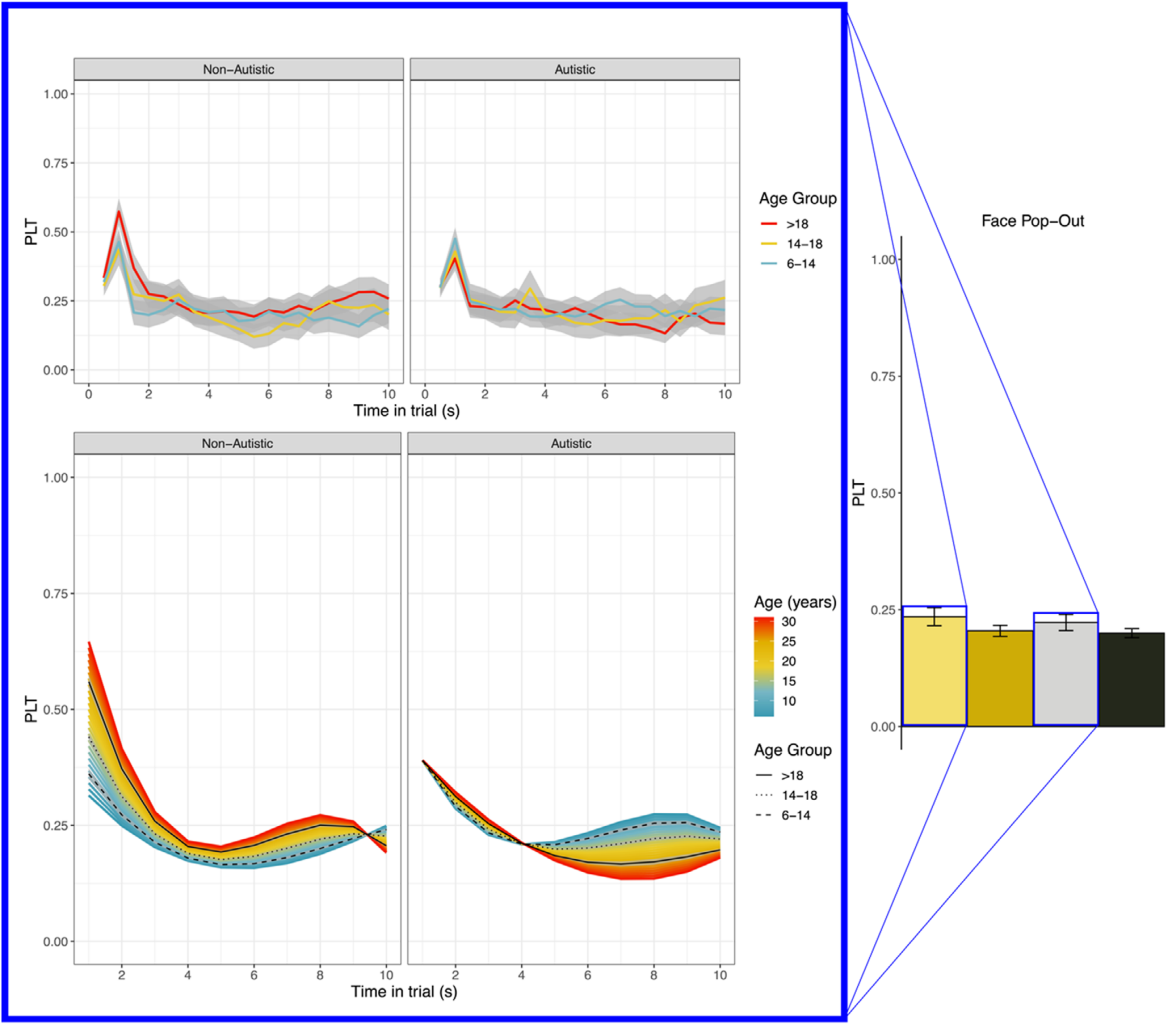
Bar plot of the intercepts – right side; representing the estimated average PLT by group – and comparisons of the raw (top left) and model estimated dynamics (bottom left) for autistic and nonautistic females in the Face Pop‐Out. We illustrated the comparison between autistic and nonautistic females because the estimates significantly differ from each other. In the left plot, the colour codes of the curves represent age, while the black lines represent the average estimate for each age group

In the nonautistic group, PLT averaged to 0.20 [0.16 ~ 0.24] (*SE* = 0.02, Table S8) and was significantly lower in males compared to females (Coef. = −0.03 [−0.05~−0.01], *SE* = 0.01; see Figure 2, right panel). In the autistic group model, PLT averaged to 0.24 [0.20 ~ 0.27] (*SE* = 0.01, Table S9), and was significantly lower in males than females (Coef. = −0.02 [−0.04~−0.006], *SE* = 0.009; see Figure 2, right panel).

##### Dynamic profile

3rd degree polynomials significantly described the change in time of PLT in both groups, configuring a transient decline (Quadratic, nonautistic: Coef. = 0.14 [0.12 ~ 0.17], *SE* = 0.01; autistic: Coef. = 0.09 [0.07 ~ 0.11], *SE* = 0.01) followed by a rise (Cubic, nonautistic: Coef. = −0.10 [−0.11~−0.08], *SE* = 0.01; autistic: Coef. = −0.07 [−0.08~−0.06], *SE* = 0.01), with a net decrease by the end of the trial (Slope, nonautistic: Coef. = −0.14 [−0.17~−0.11], *SE* = 0.01; autistic: Coef. = −0.14 [−0.16~−0.11], *SE* = 0.01). This pattern was not influenced by sex: males and females looked away from the face similarly during the course of the trial.

#### Diagnostic group differences models

##### Average profile

In the males’ model, the two diagnostic groups scored similarly on average (Table S12). In the females’ model, PLT was higher on average in the autistic compared to the nonautistic group (Coef. = 0.19 [0.12 ~ 0.24], *SE* = 0.03; Group Coef. = 0.08 [0.01 ~ 0.16], *SE* = 0.04; Table S13) but tended to decrease more with age in the autistic compared to the nonautistic group (Age Coef. = 0.08 [0.01 ~ 0.16], *SE* = 0.04; Group*Age Coef. = −0.005 [−0.01~−0.002], *SE* = 0.002; see Figure 2, left bottom panel).

##### Dynamic profile

The males did not show a definite trend of PLT throughout the trial (Slope and Cubic nonsignificant); however, a decline followed by an asymmetrical peak emerged with age (Quadratic*Age Coef. = 0.01 [0.005 ~ 0.02], *SE* = 0.003; Cubic Coeff = −0.002 [−0.005~−0.001], *SE* = 0.002); this pattern did not differ by diagnostic group. Autistic females showed a more pronounced asymmetrical, late peak compared to nonautistic females (Cubic Coef. = −0.15 [−0.25 ~ 0.02], *SE* = 0.06), but this late peak flattened with age (Cubic*Group*Age Coef. = 0.01 [0.005 ~ 0.02], *SE* = 0.003), while it increased in nonautistic females (Cubic*Age Coef. = −0.01 [−0.01~−0.002], *SE* = 0.002), such that changes were in opposite directions in the two diagnostic groups over‐development (see Figure 2, left bottom panel).

### Static scenes

#### Sex differences models

##### Average profile

In the nonautistic group, PLT averaged to 0.35 [0.22 ~ 0.46] (*SE* = 0.06; Table S16) and was marginally lower in males (Coef. = −0.02 [−0.04~−0.004], *SE* = 0.01). In the autistic group, PLT averaged to 0.39 [0.26 ~ 0.51] (*SE* = 0.05; Table S17) and was only marginally lower in males (Coef. = −0.02 [−0.04 ~ 0.003], *SE* = 0.01).

##### Dynamic profile

In the nonautistic group, 3rd degree polynomials significantly described the change in time of PLT, configuring a transient decline (Quadratic Coef. = 0.14 [0.11 ~ 0.17], *SE* = 0.02) followed by a rise (Cubic Coef. = −0.05 [−0.08~−0.03], *SE* = 0.01), with a net decrease by the end of the trial (Slope Coef. = −0.14 [0.18~−0.09], *SE* = 0.02). The 2nd degree polynomials significantly described the change in time of PLT in the autistic group, configuring a progressive decline (Quadratic Coef. = 0.12 [0.10 ~ 0.15], *SE* = 0.01), with a net decrease by the end of the trial (Slope Coef. = −0.19 [−0.22~−0.15], *SE* = 0.01). There was no significant effect of sex on the dynamic profiles.

#### Diagnostic group differences models

##### Average profile

In males, the average PLT was 0.32 [0.18 ~ 0.44] (*SE* = 0.06; Table S20), and increased with age (Coef. = 0.004 [0.002 ~ 0.01], *SE* = 0.001), but marginally less in the autistic group (Group*Age Coef. = −0.003 [−0.01 ~ 0.0001], *SE* = 0.002). In females, the average PLT was 0.35 [0.22 ~ 0.48] (*SE* = 0.07; Table S21), and increased with age (Coef. = 0.004 [0.001 ~ 0.01], *SE* = 0.002) though this was marginally less in the autistic group (Coef. = −0.005 [−0.01 ~ 0.001], *SE* = 0.002).

##### Dynamic profiles

In males, PLT marginally decreased by the end of the trial (Slope Coef. = −0.12 [−0.25~−0.02], *SE* = 0.07), with an asymmetrical U‐shape (Quadratic Coef. = 0.19 [0.07 ~ 0.30], *SE* = 0.06; Cubic Coef. = 0.10 [0.02 ~ 19], *SE* = 0.04), and a peak emerging by the end of the trial with age (Cubic*Age, Coef. = −0.01 [−0.01~−0.004], *SE* = 0.002). In females, we observe a net decrease by the end of the trial (Slope Coef. = −0.34 [−0.54~−0.10], *SE* = 0.11), approximately linear. No diagnostic group differences were significant.

### Dynamic video

#### Sex differences models

##### Average profile

**Figure 3:**
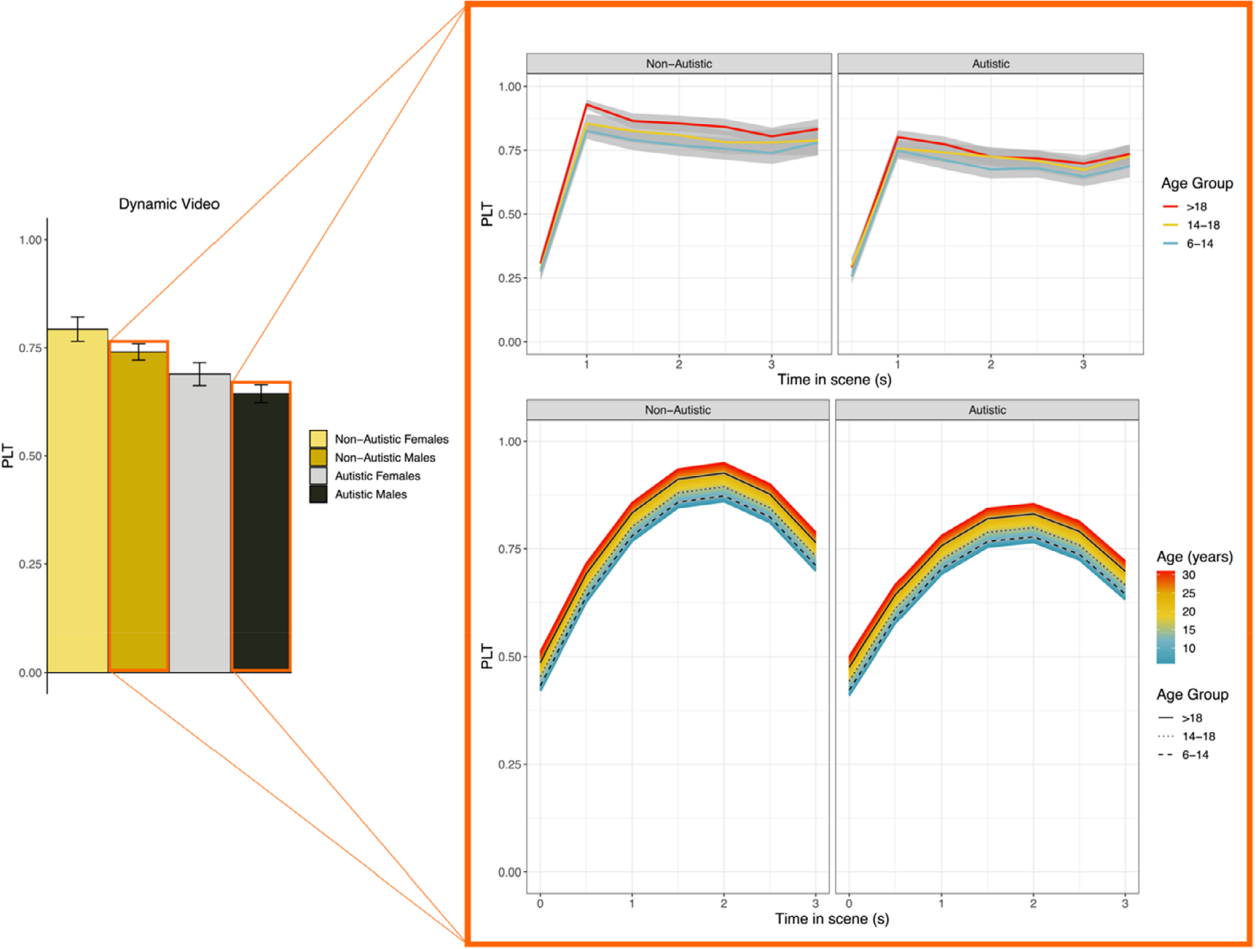
Bar plot of the intercepts – left side; representing the estimated average PLT by group – and comparisons of the raw (top right) and model estimated dynamics (bottom right) for autistic and nonautistic males in the Dynamic Video. We illustrated the comparison between autistic and nonautistic males because the estimates significantly differ from each other. In the right plot, the colour codes of the curves represent age, while the black lines represent the average estimate for each age group

In the nonautistic group, PLT averaged to 0.75 [0.68 ~ 0.82] (*SE* = 0.04; Table S24), lower in males than females (Coef. = −0.06 [−0.09~−0.03], *SE* = 0.01). In the autistic group, PLT averaged to 0.70 (*SE* = 0.04 [0.63 ~ 0.78]; Table S25), lower in males than in females (Coef. = −0.04 [−0.07~−0.004], *SE* = 0.02; see Figure 3, left panel).

##### Dynamic profile

In the nonautistic and autistic groups, the 2nd degree polynomials significantly described the changes of PLT, configuring a bell shape (nonautistic: Quadratic Coef. = −0.30 [−0.32~−0.28], *SE* = 0.01; autistic: Coef. = −0.29 [−0.32~−0.26], *SE* = 0.01), with a net increase by the end of the scene (nonautistic: Slope Coef. = 0.25 [0.23 ~ 0.27], *SE* = 0.01; autistic: Coef. = 0.22 [0.18 ~ 0.25], *SE* = 0.01). However, the bell shape flattened more in autistic males than in females (Quadratic*Sex, Coef. = 0.05 [0.02–0.09], *SE* = 0.02).

#### Diagnostic group differences models

##### Average profile

In males, the average PLT score was 0.70 [0.62 ~ 0.77] (*SE* = 0.04; Table S28) and was lower in autistic compared to nonautistic males (Coef. = −0.07 [−0.09~−0.04], *SE* = 0.01). In females, the average PLT was 0.82 [0.77 ~ 0.89] (*SE* = 0.03; Table S29) and was lower in autistic compared to nonautistic females (Coef. = −0.09 [−0.13~−0.06], *SE* = 0.02; see Figure 3, left panel).

##### Dynamic profile

In males, we saw a net increase by the end of the scene (Slope Coef. = 0.25 [0.22 ~ 0.27], *SE* = 0.01), being lower in the autistic versus nonautistic males (Coef. = −0.05 [−0.09~−0.02], *SE* = 0.02), and a bell shape (Quadratic Coef. = −0.29 [−0.31~−0.27], *SE* = 0.02), flatter in the autistic versus nonautistic males (Coef. = 0.05 [0.03 ~ 0.08], *SE* = 0.01; see Figure 3 right bottom panel). In females, we saw a net increase by the end of the scene (Slope Coef. = 0.26 [0.22 ~ 0.31], *SE* = 0.02), and a bell shape (Quadratic Coef. = −0.33 [−0.37~−0.30], *SE* = 0.02), that was marginally flatter in autistic females versus nonautistic females (Coef. = 0.04 [−0.003 ~ 0.08], *SE* = 0.02).

### Dimensional variation

In neither sex did SRS‐2 and RBS‐R relate to individual differences in Social Attention (see Appendix S1 Table S28).

In females, both the ADOS SA‐CSS (Coef. = −0.18, 95% CI = −0.28~−0.07) and the ADOS RRB‐CSS were related to the Intercept (Coef. = −0.22, 95% CI = −0.32~−0.11). In males, the relationships between Intercept and ADOS SA‐CSS (Coef. = 0.02, 95% CI = −0.05 ~ 0.08) and RRB‐CSS (Coef. = 0.02, 95% CI = −0.04 ~ 0.08) were not significant. The Quadratic Component was related to both ADOS domain scores in both females (ADOS SA‐CSS Coef. = 0.17, 95% CI = 0.07 ~ 0.27; ADOS RRB‐CSS Coef. = 0.11, 95% CI = 0.01 ~ 0.22) and males (ADOS SA‐CSS Coef. = −0.11, 95% CI = −0.17~−0.04; ADOS RRB‐CSS Coef. = −0.08, 95% CI = −0.14 ~−0.01). In both cases, the contrasts between males and females were significant (ADOS SA‐CSS, Intercept, Contr. = 0.19, *SE* = 0.06, *p*‐value = .01; Quadratic, Contr. = −0.28, *SE* = 0.06, *p*‐value < .01; ADOS RRB‐CSS, Intercept, Contr. = 0.24, *SE* = 0.06, *p*‐value < .01; Quadratic, Contr. = −0.19, *SE* = 0.06, *p*‐value = .01).

Regarding the difference score, for both intercept and quadratic, ADOS SA‐CSS had an inverse relation: when the influence of sex differences on social attention was relatively bigger than the influence of diagnostic differences, symptoms were low; i.e., females with fewer autistic symptoms tended to be more similar to neurotypical females than they were to autistic males for both linear (Coef. = −0.001, 95% CI = −0.002~−0.0008; see the full list of coefficients Appendix S1 Table S29) and quadratic aspects of social attention (Coef. = 0.0006, 95% CI = 0.00004~−0.001). In other words, when face‐looking was more influenced by sex than a diagnosis in autistic females, social‐communication symptoms were lower.

## Discussion

In this large sample of autistic and nonautistic individuals aged 6–30 years, we found that (a) females showed more social attention than males, in both autistic and nonautistic groups; (b) social attention in autistic females significantly differed from nonautistic females, but differently depending on stimulus (see below); and (c) in females, more observed autistic symptoms were associated with poorer social attention, and a greater effect for sex differences relative to diagnostic group differences on social attention was associated with fewer social‐communication symptoms.

The patterns of our findings for averaged face looking were similar to those found by Harrop et al. (2019, 2020) in that autistic females show more face‐looking than males. Another aspect that was replicated was higher average face‐looking in autistic females compared to nonautistic females in the leanest stimulus, the Face Pop‐Out; Harrop and colleagues' suggestion that autistic females’ attention may fall on a continuum (from increased for a lean scene, to decreased for a complex scene) is supported here. Furthermore, autistic females showed a later peak compared to nonautistic females, influenced by age, flattening/growing in autistic/nonautistic females respectively. As age did not influence sex differences, and autistic males did not strongly differ from nonautistic males for this stimulus, the age effect seems specific to being female and autistic. This might possibly explain the consistency with previous studies, despite the wider age range in our sample, and relate to sensitive time windows of learning in females that do not overlap across diagnostic groups.

In line with previous findings (Kaliukhovich et al., 2020; Pierce et al., 2016; Tang, Chen, Falkmer, Bölte, & Girdler, 2019), in the dynamic video, the social attention of autistic females diverged from that of nonautistic females at the average level. However, we found no difference at the dynamic level, so the evidence for a different process is less strong. In contrast, autistic males differed from nonautistic males in this context and evaded or diverted their attention from the face during the scene. This pattern may be a sign of less contextual adjustment of attention in autistic males – and since it is evident in the dynamic stimulus only, it may relate to differences in endogenous orienting that may only be elicited under more naturalistic conditions (Chevallier et al., 2015) and prolonged viewing times (Del Bianco et al., 2021). Future research could assess if this pattern is associated with specific early‐stage processing differences of attentional control, coupled with weaker activation of compensatory anterior cortical systems (Johnson et al., 2021).

Of note, we did not find a consistent difference in the Static Scenes, for which the explanation may reside in the wider Confidence Intervals (~25% in the Static Scenes, vs. <10% in the Face Pop‐Out and ~15% in the Dynamic Scenes) that indicate increased variability that may have shadowed sex and diagnostic group differences in the split samples. However, it is notable that the pattern of the diagnostic group and sex differences were consistent across all stimuli (see Table 2).

In general, this pattern of findings resonates with accounts of different levels of sensitivity and effect sizes between stimuli, interpreted as one’s social attention adapting differently to different stimuli in autistic people compared to nonautistic people. Furthermore, our findings suggest that sex adds an additional layer to the complexity, with social attention to leanest stimuli being more sensitive to differences between autistic vs. nonautistic females, and dynamic stimuli eliciting different social attention behaviour in males. It is possible that eye‐tracking captures different processes in females (e.g., adaptation and learning from simple social information) and in males (e.g., preferential looking and withdrawal with naturalistic input) that emerge dynamically from genetic predisposition and in response to social stimulation (Johnson et al., 2021).

### Dimensional variation

In females, average face‐looking was inversely associated with ADOS SA‐CSS and RRB‐CSS, meaning that females with higher social attention had lower severity scores. This pattern fits with the idea that females with a diagnosis of ASD who have higher levels of social attention may display fewer cardinal autistic symptoms across domains. It is possible that social attention may partially mitigate the effects of yet unidentified etiological mechanisms operating in the earliest postnatal developmental stages (Chawarska et al., 2016), and early‐stage processing differences (Johnson et al., 2021) that may otherwise broadly increase symptom load.

The relationship with the difference score shows that this modulation holds stronger when an individual autistic female is more different from males than she is from neurotypical females (i.e., when the effect of sex outweighs the effect of diagnostic group on social attention). This observation further supports the idea that social attention may be a manifestation of adaptation/learning in autistic females, thus *attenuating* their autistic behavioural presentation compared to that of the standard levels of males.

### Limitations

The implications of our cross‐sectional findings are limited to concurrent relationships with diagnostic status and symptomatology rather than causal inferences. Also, we did not have information about the individuals’ gender identity, gender expression, gendered socialisation experiences and other sex‐related biological factors, such as a pubertal stage. Notably, we did not find associations between eye‐tracking metrics and parent‐reported measures of autistic characteristics, which may represent a more comprehensive assay of everyday autistic presentation compared to ADOS CSS. Finally, the stimuli used, although one of them was dynamic, may not faithfully represent the experience of social attention during everyday life; the fixed order of the stimuli may not allow for disentangling the effect of stimulus order from stimulus nature – even though we did not statistically compare the three tasks to each other – and the gender imbalance (of the actor) in the SS stimuli might have influenced visual preferences.

## Conclusions

Overall, our results indicate that sex differences in social attention exist. They differ between autistic and nonautistic people, are context‐dependent and may be underpinned by multiple mechanisms depending on stimulus type. Furthermore, females with higher levels of social attention show fewer observed autistic symptoms. These observations suggest that social attention may be a candidate modifier that ameliorates autistic social‐communication characteristics by granting occasions for learning and enhancing the navigation of the human social world: for example, it may provide the autistic person with a tool for cultivating rewarding and desired social interactions, and/or avoiding unpleasant and stressful ones. Since we found associations between social attention and dimensional symptoms across domains, future work could re‐evaluate the concept of core symptoms as a continuous distribution of mild to elevated autism‐ness (Braithwaite, Gui, & Jones, 2020; Constantino, 2011). Ultimately, the modifying effect of social attention that may influence the emergence of social‐communication disability should be examined longitudinally and investigated in relationship with phenomena such as compensation and camouflaging (Lai et al., 2021).

## Supporting information


**Appendix S1**. Results.
**Table S1**. beta coefficients, standard errors (*SE*), t‐values and *p*‐values of the multiple regression with accuracy across the session.
**Table S2**. Average percentage (%) of Missing Data, and standard deviation, per time bin by stimulus, group and sex. Cohen’s D provides the effect size of the between‐sex difference within each group.
**Table S3**. Average percentage (%) of Missing Data, and standard deviation, per time bin by stimulus, group and sex. Cohen’s D provides the effect size of the between‐sex difference within each group.
**Table S4**. Non‐autistic group model selection output, comparing the base model (i.e., including polynomials of degree 3 as fixed effects and random effects), with models with additional fixed effects. ‘+’ marks adding the specified variable as a covariate; ‘*’ marks adding the interaction between the polynomial components and the specified variable. A *p*‐value < 0.05 marks a significant comparison, i.e., better explanatory power compared to the base model.
**Table S5**. Autistic group model selection output, comparing the base model (i.e., including polynomials of degree 3 as fixed effects and random effects), with models with additional fixed effects. ‘+’ marks adding the specified variable as a covariate; ‘*’ marks adding the interaction between the polynomial components and the specified variable. A *p*‐value < 0.05 marks a significant comparison, i.e., better explanatory power compared to the base model.
**Table S6**. Non‐autistic group model output, with ‘*’ marking Interactions. Significant β are marked with a *P*‐Value < 0.05, to be interpreted as different from the reference level (female).
**Table S7**. Autistic group model output, with ‘*’ marking Interactions. Significant β are marked with a *P*‐Value < 0.05, to be interpreted as different from the reference level (female).
**Table S8**. Males model selection output, comparing the base model (i.e., including polynomials of degree 3 as fixed effects and random effects), with models with additional fixed effects. ‘+’ marks adding the specified variable as a covariate; ‘*’ marks adding the interaction between the polynomial components and the specified variable. A *p*‐value < 0.05 marks a significant comparison, i.e., better explanatory power compared to the base model.
**Table S9**. Females model selection output, comparing the base model (i.e., including polynomials of degree 3 as fixed effects and random effects), with models with additional fixed effects. ‘+’ marks adding the specified variable as a covariate; ‘*’ marks adding the interaction between the polynomial components and the specified variable. A *p*‐value < 0.05 marks a significant comparison, i.e., better explanatory power compared to the base model.
**Table S10**. Males model output, with ‘*’ marking Interactions. Significant β are marked with a *P*‐Value < 0.05, to be interpreted as different from the reference level (non‐autistic).
**Table S11**. Females model output, with ‘*’ marking Interactions. Significant β are marked with a *P*‐Value < 0.05, to be interpreted as different from the reference level (non‐autistic).
**Table S12**. Non‐autistic group model selection output, comparing the base model (i.e., including polynomials of degree 3 as fixed effects and random effects), with models with additional fixed effects. ‘+’ marks adding the specified variable as a covariate; ‘*’ marks adding the interaction between the polynomial components and the specified variable. A *p*‐value < 0.05 marks a significant comparison, i.e., better explanatory power compared to the base model.
**Table S13**. Autistic group model selection output, comparing the base model (i.e., including polynomials of degree 3 as fixed effects and random effects), with models with additional fixed effects. ‘+’ marks adding the specified variable as a covariate; ‘*’ marks adding the interaction between the polynomial components and the specified variable. A *p*‐value < 0.05 marks a significant comparison, i.e., better explanatory power compared to the base model.
**Table S14** Non‐autistic model output, with ‘*’ marking Interactions. Significant β are marked with a *P*‐Value < 0.05, to be interpreted as different from the reference level (female).
**Table S15**. Autistic group model output, with ‘*’ marking Interactions. Significant β are marked with a *P*‐Value < 0.05, to be interpreted as different from the reference level (female).
**Table S16**. Males model selection output, comparing the base model (i.e., including polynomials of degree 3 as fixed effects and random effects), with models with additional fixed effects. ‘+’ marks adding the specified variable as a covariate; ‘*’ marks adding the interaction between the polynomial components and the specified variable. A *p*‐value < 0.05 marks a significant comparison, i.e., better explanatory power compared to the base model.
**Table S17**. Females model selection output, comparing the base model (i.e., including polynomials of degree 3 as fixed effects and random effects), with models with additional fixed effects. ‘+’ marks adding the specified variable as a covariate; ‘*’ marks adding the interaction between the polynomial components and the specified variable. A *p*‐value < 0.05 marks a significant comparison, i.e., better explanatory power compared to the base model.
**Table S18**. Males model output, with ‘*’ marking Interactions. Significant β are marked with a *P*‐Value < 0.05, to be interpreted as different from the reference level (non‐autistic).
**Table S19**. Females model output, with ‘*’ marking Interactions. Significant β are marked with a *P*‐Value < 0.05, to be interpreted as different from the reference level (non‐autistic).
**Table S20**. Non‐autistic group model selection output, comparing the base model (i.e., including polynomials of degree 3 as fixed effects and random effects), with models with additional fixed effects. ‘+’ marks adding the specified variable as a covariate; ‘*’ marks adding the interaction between the polynomial components and the specified variable. A *p*‐value < 0.05 marks a significant comparison, i.e., better explanatory power compared to the base model.
**Table S21**. Autistic group model selection output, comparing the base model (i.e., including polynomials of degree 3 as fixed effects and random effects), with models with additional fixed effects. ‘+’ marks adding the specified variable as a covariate; ‘*’ marks adding the interaction between the polynomial components and the specified variable. A *p*‐value < 0.05 marks a significant comparison, i.e., better explanatory power compared to the base model.
**Table S22**. Non‐autistic group model output, with ‘*’ marking Interactions. Significant β are marked with a *P*‐Value < 0.05, to be interpreted as different from the reference level (female).
**Table S23**. Autistic group model output, with ‘*’ marking Interactions. Significant β are marked with a *P*‐Value < 0.05, to be interpreted as different from the reference level (female).
**Table S24**. Males model selection output, comparing the base model (i.e., including polynomials of degree 3 as fixed effects and random effects), with models with additional fixed effects. ‘+’ marks adding the specified variable as a covariate; ‘*’ marks adding the interaction between the polynomial components and the specified variable. A *p*‐value < 0.05 marks a significant comparison, i.e., better explanatory power compared to the base model.
**Table S25**. Females model selection, comparing the base model (i.e., including polynomials of degree 3 as fixed effects and random effects), with models with additional fixed effects. ‘+’ marks adding the specified variable as a covariate; ‘*’ marks adding the interaction between the polynomial components and the specified variable. A *p*‐value < 0.05 marks a significant comparison, i.e., better explanatory power compared to the base model.
**Table S26**. Males model output, with ‘*’ marking Interactions. Significant β are marked with a *P*‐Value < 0.05, to be interpreted as different from the reference level (female).
**Table S27**. Females model output, with ‘*’ marking Interactions. Significant β are marked with a *P*‐Value < 0.05, to be interpreted as different from the reference level (female).
**Table S8**. Contrasts specification, with coefficient estimate of the slope (significant coefficients marked with ‘*’), and the difference to which the statistical test has been applied.
**Table S29**. Difference score/ADOS SA‐CSS multiple linear regression (significant coefficients marked with ‘*’).

## Appendix

The EU‐AIMS LEAP Group: Antonia San Jose Caceres, Daisy Crawley, Jessica Faulkner, Jessica Sabet, Claire Ellis, Bethany Oakley, Rosemary Holt, Sara Ambrosino, Nico Bast, Sarah Baumeister, Annika Rausch, Carsten Bours, Ineke Cornelissen, Daniel von Rhein, Larry O’Dwyer, Jumana Ahmad, Emily Simonoff, Sarah Durston, Tobias Banaschewski, Andrea Persico
