# Supplementary material for: Unique dynamic profiles of social attention in autistic females
Source: J Child Psychol Psychiatry. 2022 May 30;63(12):1602–14. doi: 10.1111/jcpp.13630 (PMC9796530; doi:10.1111/jcpp.13630)
Supplement: Supplementary file 1 — Appendix S1. Results. Table S1. beta coefficients, standard errors (SE), t‐values and p‐values of the multiple regression with accuracy across the session. Table S2. Average percentage (%) of Missing Data, and standard deviation, per time bin by stimulus, group and sex. Cohen’s D provides the effect size of the between‐sex difference within each group. Table S3. Average percentage (%) of Missing Data, and standard deviation, per time bin by stimulus, group and sex. Cohen’s D provides the effect size of the between‐sex difference within each group. Table S4. Non‐autistic group model selection output, comparing the base model (i.e., including polynomials of degree 3 as fixed effects and random effects), with models with additional fixed effects. ‘+’ marks adding the specified variable as a covariate; ‘*’ marks adding the interaction between the polynomial components and the specified variable. A p‐value < 0.05 marks a significant comparison, i.e., better explanatory power compared to the base model. Table S5. Autistic group model selection output, comparing the base model (i.e., including polynomials of degree 3 as fixed effects and random effects), with models with additional fixed effects. ‘+’ marks adding the specified variable as a covariate; ‘*’ marks adding the interaction between the polynomial components and the specified variable. A p‐value < 0.05 marks a significant comparison, i.e., better explanatory power compared to the base model. Table S6. Non‐autistic group model output, with ‘*’ marking Interactions. Significant β are marked with a P‐Value < 0.05, to be interpreted as different from the reference level (female). Table S7. Autistic group model output, with ‘*’ marking Interactions. Significant β are marked with a P‐Value < 0.05, to be interpreted as different from the reference level (female). Table S8. Males model selection output, comparing the base model (i.e., including polynomials of degree 3 as fixed effects and random effects), with models with [file JCPP-63-1602-s001.docx]

Supporting Information

# Appendix S1. Results

This section includes results of the analyses that, for space limits reasons, could not be included in the main text. Each heading indicates the dependent variable of the analysis in question.

## S1.1 Accuracy

Since the task was part of a 28-minutes battery including other tasks, we calculated the accuracy of the calibration across the whole session, as a quality-check of the data over time. To investigate differences in the accuracy of the eye-tracking across the whole session, we used a multiple linear regression with Accuracy as dependent variable, and group in interaction with age as predictors. We found no differences by group/age, see Table S1 for a full report of the coefficients, standard errors, and p-values.

*Table S1: beta coefficients, standard errors (SE), t-values and p-values of the multiple regression with accuracy across the session*

| Term | Coefficient | SE | t-value | p-value |
| --- | --- | --- | --- | --- |
| Intercept | 0.0486 | 0.0020 | 24.12 | <0.001 |
| Group: Autistic | 0.0019 | 0.0025 | 0.75 | 0.44 |
| Age Group: 14-18 | 0.0032 | 0.0030 | 1.06 | 0.28 |
| Age Group: >18 | 0.0035 | 0.0027 | 1.29 | 0.19 |
| Group: Autistic * Age Group: 14-18 | -0.0012 | 0.0039 | -0.31 | 0.75 |
| Group: Autistic * Age Group: >18 | -0.0013 | 0.0035 | -0.36 | 0.71 |

## S1.2 Missing Data

Given that the % of missing data differed between groups by diagnosis and sex across stimuli, we calculated effect sizes (Cohen’s D) for each comparison, to evaluate whether this could be attributed to systematic or random variability. All comparisons concluded small effect sizes (<0.4; Table S3-4), suggesting differences may be due to idiosyncratic differences. We treated this imbalance by assuming % of missing data as a covariate in all analytical models.

*Table S2: Average percentage (%) of Missing Data, and standard deviation, per time bin by stimulus, group and sex. Cohen’s D provides the effect size of the between-sex difference within each group.*

| Stimulus | Group | Sex | % Missing (SD) | Cohen’s D |
| --- | --- | --- | --- | --- |
| Face Pop-Out | Autistic | Female | 7.08 (15.28) | 0.03 |
|  |  | Male | 6.49 (14.63) |  |
|  | Non-autistic | Female | 5.36 (13.45) | 0.008 |
|  |  | Male | 5.47 (13.70) |  |
| Static Scenes | Autistic | Female | 9.06 (17.27) | 0.02 |
|  |  | Male | 9.54 (17.87) |  |
|  | Non-autistic | Female | 7.05 (15.78) | 0.009 |
|  |  | Male | 7.20 (15.75) |  |
| Dynamic Video | Autistic | Female | 8.42 (13.21) | 0.04 |
|  |  | Male | 9.06 (14.04) |  |
|  | Non-autistic | Female | 5.21 (10.90) | 0.04 |
|  |  | Male | 4.76 (9.37) |  |

*Table S3: Average percentage (%) of Missing Data, and standard deviation, per time bin by stimulus, group and sex. Cohen’s D provides the effect size of the between-sex difference within each group.*

| Stimulus | Sex | Sex | Mean % (SD) | Cohen’s D |
| --- | --- | --- | --- | --- |
| Face Pop-Out | Female | Autistic | 7.08 (15.28) | 0.11 |
|  |  | Non-autistic | 5.36 (13.45) |  |
|  | Male | Autistic | 6.49 (14.63) | 0.07 |
|  |  | Non-autistic | 5.47 (13.70) |  |
| Static Scenes | Female | Autistic | 9.06 (17.27) | 0.11 |
|  |  | Non-autistic | 7.05 (15.78) |  |
|  | Male | Autistic | 9.54 (17.87) | 0.13 |
|  |  | Non-autistic | 7.20 (15.75) |  |
| Dynamic Video | Female | Autistic | 8.42 (13.21) | 0.24 |
|  |  | Non-autistic | 5.21 (10.90) |  |
|  | Male | Autistic | 9.06 (14.04) | 0.34 |
|  |  | Non-autistic | 4.76 (9.37) |  |

## S1.3 Growth Curve Analysis

Below we report the output of the Likelihood Ratio tests performed to ensure the best model fit, indicated by significant p-values.

### S1.3.1 Face Pop-Out

#### S1.3.1.1 Sex Differences Model Selection

For the non-autistic group, the model including 3rd degree polynomials, sex, age, and the proportion of missing data per time bin as covariates proved the best fit compared to the base model (Table S4). For the autistic group, the model including sex and the proportion of missing data as covariates of 3rd degree polynomials proved the best fit compared to the base model (Table S5).

*Table S4****: Non-autistic group model selection output****, comparing the base model (i.e., including polynomials of degree 3 as fixed effects and random effects), with models with additional fixed effects. ‘+’ marks adding the specified variable as a covariate; ‘*’ marks adding the interaction between the polynomial components and the specified variable. A p-value < 0.05 marks a significant comparison, i.e., better explanatory power compared to the base model.*

| Group | Model | N Parameters | AIC | BIC | Deviance | Statistic | DF | P-value |
| --- | --- | --- | --- | --- | --- | --- | --- | --- |
| Non-autistic | Base | 12 | 24991.60 | 25094.25 | 24967.60 | - | - | - |
|  | + Sex | 13 | 24985.80 | 25097.01 | 24959.80 | 7.80 | 1 | 0.01 |
|  | * Sex | 16 | 24986.76 | 25123.64 | 24954.76 | 5.04 | 3 | 0.17 |
|  | + Sex + Age | 14 | 24974.51 | 25094.28 | 24946.51 | 13.29 | 1 | <0.001 |
|  | + Sex * Age | 15 | 24975.92 | 25104.24 | 24945.92 | 0.59 | 1 | 0.44 |
|  | + Sex + Age + Proportion of Missing Values | 15 | 24684.37 | 24812.69 | 24654.37 | 292.14 | 1 | <0.001 |

*Table S5:* ***Autistic group model selection output****, comparing the base model (i.e., including polynomials of degree 3 as fixed effects and random effects), with models with additional fixed effects. ‘+’ marks adding the specified variable as a covariate; ‘*’ marks adding the interaction between the polynomial components and the specified variable. A p-value < 0.05 marks a significant comparison, i.e., better explanatory power compared to the base model.*

| Group | Model | N Parameters | AIC | BIC | Deviance | Statistic | DF | P-value |
| --- | --- | --- | --- | --- | --- | --- | --- | --- |
| Autistic | Base | 12 | 30837.27 | 30943.46 | 30813.27 | - | - | - |
|  | + Sex | 13 | 30833.38 | 30948.42 | 30807.38 | 5.89 | 1 | 0.02 |
|  | * Sex | 16 | 30834.19 | 30975.77 | 30802.19 | 5.20 | 3 | 0.16 |
|  | + Sex + Age | 14 | 30835.38 | 30959.26 | 30807.38 | 0.01 | 1 | 0.93 |
|  | + Sex * Age | 15 | 30834.78 | 30967.52 | 30804.78 | 2.59 | 1 | 0.11 |
|  | + Sex + Proportion of Missing Values | 14 | 30419.27 | 30543.15 | 30391.27 | 416.12 | 1 | <0.001 |

#### S1.3.1.2 Sex Differences Model Complete Output

In addition to the report of the significant coefficients in the main text, we report below the list of all coefficients and measures (Table S7 and S8).

*Table S6:* ***Non-autistic group model output****, with ‘*’ marking Interactions. Significant β are marked with a P-Value < 0.05, to be interpreted as different from the reference level (female).*

| Group | Term | Coef. | SE | DF | T-value | P-Value | CI 2.5% | CI 97.5% | χ^2^(1), p-value |
| --- | --- | --- | --- | --- | --- | --- | --- | --- | --- |
| Non-autistic | Intercept | 0.20 | 0.02 | 46.17 | 9.60 | <0.001 | 0.16 | 0.24 | 92.16, <0.01 |
|  | Slope | -0.14 | 0.01 | 273.43 | -9.60 | <0.001 | -0.17 | -0.11 | 92.21, <0.01 |
|  | Quadratic Component | 0.14 | 0.01 | 272.07 | 11.61 | <0.001 | 0.12 | 0.17 | 134.88, <0.01 |
|  | Cubic Component | -0.10 | 0.01 | 37640.37 | -12.27 | <0.001 | -0.11 | -0.08 | 150.63, <0.01 |
|  | Sex | -0.03 | 0.01 | 269.21 | -3.17 | 0.002 | -0.05 | -0.01 | 10.03, <0.01 |
|  | Age | 0.003 | 0.001 | 269.79 | 3.73 | <0.001 | 0.001 | 0.005 | 13.90, <0.01 |
|  | Proportion of Missing Data | -0.23 | 0.01 | 37240.45 | -17.13 | <0.001 | -0.25 | -0.20 | 293.44, <0.01 |

*Table S7:* ***Autistic group model output****, with ‘*’ marking Interactions. Significant β are marked with a P-Value < 0.05, to be interpreted as different from the reference level (female).*

| Group | Term | Coef. | SE | DF | T-value | P-Value | CI 2.5% | CI 97.5% | χ^2^(1), p-value |
| --- | --- | --- | --- | --- | --- | --- | --- | --- | --- |
| Autistic | Intercept | 0.24 | 0.01 | 13.20 | 17.07 | <0.001 | 0.20 | 0.27 | 291.35, <0.01 |
|  | Slope | -0.14 | 0.01 | 393.42 | -11.85 | <0.001 | -0.16 | -0.11 | 140.54, <0.01 |
|  | Quadratic Component | 0.09 | 0.01 | 381.63 | 8.17 | <0.001 | 0.07 | 0.11 | 66.68, <0.01 |
|  | Cubic Component | -0.07 | 0.01 | 50544.37 | -10.10 | <0.001 | -0.08 | -0.06 | 102.01, <0.01 |
|  | Sex | -0.02 | 0.01 | 382.67 | -2.68 | 0.02 | -0.04 | -0.006 | 7.21, <0.01 |
|  | Proportion of Missing Data | -0.21 | 0.01 | 49974.51 | -20.46 | <0.001 | -0.23 | -0.19 | 418.56, <0.01 |

#### S1.3.1.3 Diagnostic Groups Model Selection

For both models (males and females), the model including diagnosis and age in interaction with 3rd degree polynomials, and the proportion of missing data per time bin as covariate obtained the best fit (Table S8 and S9).

*Table S8:* ***Males model selection output****, comparing the base model (i.e., including polynomials of degree 3 as fixed effects and random effects), with models with additional fixed effects. ‘+’ marks adding the specified variable as a covariate; ‘*’ marks adding the interaction between the polynomial components and the specified variable. A p-value < 0.05 marks a significant comparison, i.e., better explanatory power compared to the base model.*

| Sex | Model | N Parameters | AIC | BIC | Deviance | Statistic | DF | P-value |
| --- | --- | --- | --- | --- | --- | --- | --- | --- |
| Male | Base | 12 | 36866.83 | 36975.33 | 36842.83 | NA | - | - |
|  | + Group | 13 | 36868.34 | 36985.89 | 36842.34 | 0.49 | 1 | 0.49 |
|  | * Group | 16 | 36864.61 | 37009.28 | 36832.61 | 9.74 | 3 | 0.02 |
|  | * Group + Age | 17 | 36860.31 | 37014.02 | 36826.31 | 6.30 | 1 | 0.01 |
|  | * Group * Age | 24 | 36826.50 | 37043.51 | 36778.50 | 47.81 | 7 | <0.001 |
|  | * Group * Age + Proportion of Missing Data | 25 | 36317.59 | 36543.63 | 36267.59 | 510.92 | 1 | <0.001 |

*Table S9:* ***Females model selection output****, comparing the base model (i.e., including polynomials of degree 3 as fixed effects and random effects), with models with additional fixed effects. ‘+’ marks adding the specified variable as a covariate; ‘*’ marks adding the interaction between the polynomial components and the specified variable. A p-value < 0.05 marks a significant comparison, i.e., better explanatory power compared to the base model.*

| Sex | Model | N Parameters | AIC | BIC | Deviance | Statistic | DF | P-value |
| --- | --- | --- | --- | --- | --- | --- | --- | --- |
| Female | Base | 12 | 18892.23 | 18990.86 | 18868.23 | - | - | - |
|  | + Group | 13 | 18893.08 | 18999.94 | 18867.08 | 1.15 | 1 | 0.28 |
|  | * Group | 16 | 18889.25 | 19020.76 | 18857.25 | 9.83 | 3 | 0.02 |
|  | * Group + Age | 17 | 18890.38 | 19030.11 | 18856.38 | 0.87 | 1 | 0.35 |
|  | * Group * Age | 24 | 18873.37 | 19070.63 | 18825.37 | 31.01 | 7 | <0.001 |
|  | * Group * Age + Proportion of Missing Data | 25 | 18678.56 | 18884.04 | 18628.56 | 196.81 | 1 | <0.001 |

#### S1.3.1.4 Diagnostic Groups Model Output

In addition to the report of the significant coefficients in the main text, we report below the list of all coefficients and measures (Tables S10 and S11).

*Table S10:* ***Males model output****, with ‘*’ marking Interactions. Significant β are marked with a P-Value < 0.05, to be interpreted as different from the reference level (non-autistic).*

| Sex | Term | Coef. | SE | DF | T-value | P-Value | CI 2.5% | CI 97.5% | χ^2^(1), p-value |
| --- | --- | --- | --- | --- | --- | --- | --- | --- | --- |
| Male | Intercept | 0.19 | 0.02 | 71.60 | 8.54 | <0.001 | 0.15 | 0.23 | 72.99, <0.01 |
|  | Slope | -0.01 | 0.05 | 459.85 | -0.21 | 0.83 | -0.12 | 0.11 | 0.04, 0.83 |
|  | Quadratic Component | -0.06 | 0.05 | 451.89 | -1.28 | 0.20 | -0.15 | 0.06 | 1.64, 0.20 |
|  | Cubic Component | -0.06 | 0.03 | 61325.88 | -2.00 | 0.05 | -0.12 | -0.01 | 3.99, 0.05 |
|  | Group | 0.02 | 0.02 | 452.86 | 0.98 | 0.33 | -0.02 | 0.08 | 0.95, 0.33 |
|  | Age | 0.002 | 0.001 | 447.71 | 1.74 | 0.08 | <0.001 | 0.004 | 3.03, 0.08 |
|  | Proportion of Missing Data | -0.22 | 0.01 | 61111.82 | -22.66 | <0.001 | -0.24 | -0.20 | 513.29, <0.01 |
|  | Slope * Group | -0.07 | 0.07 | 464.34 | -1.09 | 0.28 | -0.22 | 0.04 | 1.18, 0.28 |
|  | Quadratic * Group | 0.01 | 0.06 | 456.56 | 0.15 | 0.88 | -0.11 | 0.13 | 0.02, 0.88 |
|  | Cubic * Group | 0.05 | 0.04 | 61322.93 | 1.28 | 0.20 | -0.01 | 0.12 | 1.64, 0.20 |
|  | Slope * Age | -0.01 | 0.003 | 453.39 | -2.53 | 0.01 | -0.01 | -0.002 | 6.39, 0.01 |
|  | Quadratic * Age | 0.01 | 0.003 | 444.31 | 4.24 | <0.001 | 0.005 | 0.02 | 17.95, <0.01 |
|  | Cubic * Age | -0.002 | 0.002 | 61262.19 | -1.41 | 0.16 | -0.005 | 0.001 | 1.98, 0.16 |
|  | Group * Age | -0.002 | 0.001 | 450.03 | -1.11 | 0.26 | -0.005 | 0.001 | 1.24, 0.26 |
|  | Slope * Group * Age | 0.004 | 0.004 | 458.55 | 0.95 | 0.34 | -0.003 | 0.01 | 0.90, 0.34 |
|  | Quadratic * Group * Age | -0.003 | 0.003 | 449.80 | -0.87 | 0.38 | -0.01 | 0.004 | 0.76, 0.38 |
|  | Cubic * Group * Age | -0.002 | 0.002 | 61271.37 | -0.75 | 0.45 | -0.01 | 0.002 | 0.56, 0.45 |

*Table S11****: Females model output****, with ‘*’ marking Interactions. Significant β are marked with a P-Value < 0.05, to be interpreted as different from the reference level (non-autistic).*

| Sex | Term | Coef. | SE | DF | T-value | P-Value | CI 2.5% | CI 97.5% | χ^2^(1), p-value |
| --- | --- | --- | --- | --- | --- | --- | --- | --- | --- |
| Female | Intercept | 0.19 | 0.03 | 135.64 | 5.89 | <0.001 | 0.125 | 0.247 | 34.71, <0.01 |
|  | Slope | 0.003 | 0.08 | 196.29 | 0.04 | 0.97 | -0.164 | 0.154 | <0.01, 0.97 |
|  | Quadratic Component | 0.12 | 0.07 | 193.28 | 1.70 | 0.09 | -0.027 | 0.243 | 2.86, 0.09 |
|  | Cubic Component | 0.03 | 0.04 | 26910.19 | 0.64 | 0.52 | -0.07 | 0.12 | 0.41, 0.52 |
|  | Group | 0.08 | 0.04 | 197.58 | 2.20 | 0.03 | 0.01 | 0.16 | 4.86, 0.03 |
|  | Age | 0.004 | 0.002 | 197.76 | 2.16 | 0.03 | <0.001 | 0.006 | 4.65, 0.03 |
|  | Proportion of Missing Data | -0.22 | 0.01 | 25992.45 | -14.07 | <0.001 | -0.25 | -0.19 | 197.99, <0.01 |
|  | Slope * Group | -0.02 | 0.10 | 198.53 | -0.22 | 0.83 | -0.22 | 0.21 | 0.05, 0.83 |
|  | Quadratic * Group | -0.02 | 0.09 | 195.80 | -0.18 | 0.86 | -0.18 | 0.15 | 0.03, 0.86 |
|  | Cubic * Group | -0.15 | 0.06 | 26917.65 | -2.65 | 0.008 | -0.25 | -0.03 | 7.05, 0.01 |
|  | Slope * Age | -0.01 | 0.004 | 198.54 | -2.11 | 0.04 | -0.02 | 0.001 | 4.45, 0.03 |
|  | Quadratic * Age | 0.004 | 0.004 | 195.46 | 0.99 | 0.32 | -0.00 | 0.01 | 0.99, 0.32 |
|  | Cubic * Age | -0.01 | 0.002 | 26941.66 | -3.03 | 0.002 | -0.01 | -0.002 | 9.20, <0.01 |
|  | Group * Age | -0.005 | 0.002 | 198.09 | -2.53 | 0.01 | -0.01 | -0.002 | 6.41, 0.01 |
|  | Slope * Group * Age | 0.003 | 0.01 | 199.47 | 0.47 | 0.64 | -0.01 | 0.01 | 0.22, 0.64 |
|  | Quadratic * Group * Age | -0.002 | 0.005 | 196.70 | -0.49 | 0.63 | -0.01 | 0.007 | 0.24, 0.63 |
|  | Cubic * Group * Age | 0.01 | 0.003 | 26936.96 | 3.61 | <0.001 | 0.005 | 0.02 | 13.02, <0.01 |

### S1.3.2 Static Social Scenes

#### S1.3.2.1 Sex Differences Models

For the non-autistic group, the model including sex, age, and the proportion of missing data per time bin as covariates of 3rd degree polynomials proved the best fit (Table S12). For the autistic group, the model including sex and the proportion of missing data as covariates and 3rd degree polynomials proved the best fit (Table S13).

*Table S12:* ***Non-autistic group model selection output****, comparing the base model (i.e., including polynomials of degree 3 as fixed effects and random effects), with models with additional fixed effects. ‘+’ marks adding the specified variable as a covariate; ‘*’ marks adding the interaction between the polynomial components and the specified variable. A p-value < 0.05 marks a significant comparison, i.e., better explanatory power compared to the base model.*

| Group | Model | N Parameters | AIC | BIC | Deviance | Statistic | DF | P-value |
| --- | --- | --- | --- | --- | --- | --- | --- | --- |
| Non-autistic | Base | 12 | 54399.46 | 54507.32 | 54375.46 | - | - | - |
|  | + Sex | 13 | 54398.00 | 54514.85 | 54372.00 | 3.46 | 1 | 0.06 |
|  | * Sex | 16 | 54398.88 | 54542.68 | 54366.88 | 5.13 | 3 | 0.16 |
|  | + Sex + Age | 14 | 54381.52 | 54507.36 | 54353.52 | 18.48 | 1 | <0.001 |
|  | + Sex * Age | 15 | 54383.08 | 54517.90 | 54353.08 | 0.45 | 1 | 0.50 |
|  | + Sex + Age + Proportion of Missing Data | 15 | 54303.33 | 54438.15 | 54273.33 | 80.19 | 1 | <0.001 |

*Table S13:* ***Autistic group model selection output****, comparing the base model (i.e., including polynomials of degree 3 as fixed effects and random effects), with models with additional fixed effects. ‘+’ marks adding the specified variable as a covariate; ‘*’ marks adding the interaction between the polynomial components and the specified variable. A p-value < 0.05 marks a significant comparison, i.e., better explanatory power compared to the base model.*

| Group | Model | N Parameters | AIC | BIC | Deviance | Statistic | DF | P-value |
| --- | --- | --- | --- | --- | --- | --- | --- | --- |
| Autistic | Base | 12 | 68892.31 | 69003.59 | 68868.31 | - | - | - |
|  | + Sex | 13 | 68890.98 | 69011.52 | 68864.98 | 3.34 | 1 | 0.07 |
|  | * Sex | 16 | 68893.69 | 69042.06 | 68861.69 | 3.28 | 3 | 0.35 |
|  | + Sex + Age | 14 | 68891.71 | 69021.53 | 68863.71 | 1.26 | 1 | 0.26 |
|  | + Sex + Age | 15 | 68893.39 | 69032.49 | 68863.39 | 0.32 | 1 | 0.57 |
|  | + Sex + Proportion of Missing Data | 14 | 68766.68 | 68896.50 | 68738.68 | 126.30 | 1 | <0.01 |

#### S1.3.2.2 Sex Differences Model Output

In addition to the report of the significant coefficients in the main text, we report below the list of all coefficients and measures (Table S15 and S16).

*Table S14:* ***Non-autistic model output****, with ‘*’ marking Interactions. Significant β are marked with a P-Value < 0.05, to be interpreted as different from the reference level (female).*

| Group | Term | Coef. | SE | DF | T-value | P-Value | CI 2.5% | CI 97.5% | χ^2^(1), p-value |
| --- | --- | --- | --- | --- | --- | --- | --- | --- | --- |
| Non-autistic | Intercept | 0.35 | 0.06 | 6.03 | 5.83 | 0.001 | 0.22 | 0.46 | 34.04,  <0.01 |
|  | Slope | -0.14 | 0.02 | 263.49 | -7.22 | <0.001 | -0.18 | -0.09 | 52.16,  <0.01 |
|  | Quadratic Component | 0.14 | 0.02 | 278.35 | 9.00 | <0.001 | 0.11 | 0.17 | 81.04,  <0.01 |
|  | Cubic Component | -0.05 | 0.01 | 58647.62 | -5.04 | <0.001 | -0.08 | -0.03 | 25.44,  <0.01 |
|  | Sex | -0.02 | 0.01 | 271.84 | -1.96 | 0.05 | -0.04 | -0.004 | 3.84,  0.05 |
|  | Age | 0.004 | 0.001 | 273.48 | 4.12 | <0.001 | 0.002 | 0.005 | 16.98,  <0.01 |
|  | Proportion of Missing Data | -0.001 | <0.001 | 56188.98 | -8.96 | <0.001 | -0.001 | -0.001 | 80.29  <0.01 |

*Table S15:* ***Autistic group model output****, with ‘*’ marking Interactions. Significant β are marked with a P-Value < 0.05, to be interpreted as different from the reference level (female).*

| Group | Term | Coef. | SE | DF | T-value | P-Value | CI 2.5% | CI 97.5% | χ^2^(1), p-value |
| --- | --- | --- | --- | --- | --- | --- | --- | --- | --- |
| Autistic | Intercept | 0.39 | 0.05 | 5.33 | 7.10 | 0.001 | 0.26 | 0.51 | 50.42,  <0.01 |
|  | Slope | -0.19 | 0.01 | 368.59 | -12.63 | <0.001 | -0.22 | -0.15 | 159.52,  <0.01 |
|  | Quadratic Component | 0.12 | 0.01 | 385.10 | 9.06 | <0.001 | 0.10 | 0.15 | 82.09,  <0.01 |
|  | Cubic Component | -0.01 | 0.009 | 78006.87 | -1.57 | 0.12 | -0.03 | 0.004 | 2.46,  0.11 |
|  | Sex | -0.02 | 0.01 | 382.99 | -1.82 | 0.07 | -0.05 | 0.003 | 3.33,  0.06 |
|  | Age | -0.001 | <0.001 | 76483.68 | -11.25 | <0.001 | -0.001 | -0.001 | 126.46,  <0.01 |

#### S1.3.2.3 Diagnostic Groups Model Selection

For both males and females, the models including diagnosis and age in interaction with the 3rd degree polynomials, and the proportion of missing data per time bin as covariate obtained the best fit (Tables S16 and S17).

*Table S16:* ***Males model selection output****, comparing the base model (i.e., including polynomials of degree 3 as fixed effects and random effects), with models with additional fixed effects. ‘+’ marks adding the specified variable as a covariate; ‘*’ marks adding the interaction between the polynomial components and the specified variable. A p-value < 0.05 marks a significant comparison, i.e., better explanatory power compared to the base model.*

| Sex | Model | N Parameters | AIC | BIC | Deviance | Statistic | DF | P-value |
| --- | --- | --- | --- | --- | --- | --- | --- | --- |
| Male | Base | 12 | 84917.54 | 85031.16 | 84893.54 | - | - | - |
|  | + Group | 13 | 84913.10 | 85036.19 | 84887.10 | 6.45 | 1 | 0.01 |
|  | * Group | 16 | 84908.57 | 85060.07 | 84876.57 | 10.52 | 3 | 0.01 |
|  | * Group + Age | 17 | 84899.55 | 85060.51 | 84865.55 | 11.03 | 1 | <0.01 |
|  | * Group *Age | 24 | 84887.98 | 85115.23 | 84839.98 | 25.56 | 7 | <0.01 |
|  | * Group *Age * Proportion of Missing Data | 25 | 84748.24 | 84984.95 | 84698.24 | 141.75 | 1 | <0.01 |

*Table S17:* ***Females model selection output****, comparing the base model (i.e., including polynomials of degree 3 as fixed effects and random effects), with models with additional fixed effects. ‘+’ marks adding the specified variable as a covariate; ‘*’ marks adding the interaction between the polynomial components and the specified variable. A p-value < 0.05 marks a significant comparison, i.e., better explanatory power compared to the base model.*

| Sex | Model | N Parameters | AIC | BIC | Deviance | Statistic | DF | P-value |
| --- | --- | --- | --- | --- | --- | --- | --- | --- |
| Female | Base | 12 | 38366.25 | 38470.04 | 38342.25 | - | - | - |
|  | + Group | 13 | 38365.08 | 38477.52 | 38339.08 | 3.17 | 1 | 0.07 |
|  | * Group | 16 | 38367.54 | 38505.93 | 38335.54 | 3.54 | 3 | 0.32 |
|  | * Group + Age | 17 | 38368.18 | 38515.22 | 38334.18 | 1.36 | 1 | 0.24 |
|  | * Group *Age | 24 | 38364.53 | 38572.11 | 38316.53 | 17.65 | 7 | 0.01 |
|  | * Group *Age * Proportion of Missing Data | 25 | 38302.27 | 38518.50 | 38252.27 | 64.26 | 1 | <0.01 |

#### S1.3.2.4 Diagnostic Groups Model Output

In addition to the report of the significant coefficients in the main text, we report below the list of all coefficients and measures (Table S18 and S19).

*Table S18:* ***Males model output****, with ‘*’ marking Interactions. Significant β are marked with a P-Value < 0.05, to be interpreted as different from the reference level (non-autistic).*

| Sex | Term | Coef. | SE | DF | T-value | P-Value | CI 2.5% | CI 97.5% | χ^2^(1), p-value |
| --- | --- | --- | --- | --- | --- | --- | --- | --- | --- |
| Male | Intercept | 0.32 | 0.06 | 7.09 | 5.47 | 0.001 | 0.18 | 0.44 | 29.92,  <0.01 |
|  | Slope | -0.12 | 0.07 | 435.89 | -1.79 | 0.07 | -0.25 | 0.02 | 3.21,  0.07 |
|  | Quadratic Component | 0.19 | 0.06 | 458.30 | 3.15 | 0.002 | 0.07 | 0.30 | 9.90,  <0.01 |
|  | Cubic Component | 0.10 | 0.04 | 94843.57 | 2.49 | 0.01 | 0.02 | 0.19 | 6.22,  0.01 |
|  | Group | 0.03 | 0.03 | 454.72 | 0.93 | 0.35 | -0.03 | 0.08 | 0.86,  0.35 |
|  | Age | 0.004 | 0.001 | 448.39 | 3.22 | 0.001 | 0.002 | 0.01 | 10.35,  <0.01 |
|  | Proportion of Missing Data | -0.001 | <0.001 | 93984.79 | -11.91 | <0.001 | -0.001 | -0.001 | 141.87,  <0.01 |
|  | Slope * Group | -0.09 | 0.09 | 443.02 | -1.03 | 0.30 | -0.26 | 0.09 | 1.06,  0.30 |
|  | Quadratic Component * Group | -0.08 | 0.08 | 469.50 | -1.01 | 0.31 | -0.25 | 0.09 | 1.03,  0.31 |
|  | Cubic Component * Group | -0.01 | 0.05 | 94703.43 | -0.17 | 0.86 | -0.14 | 0.09 | 0.03,  0.86 |
|  | Slope * Age | <0.001 | 0.004 | 426.77 | -0.11 | 0.91 | -0.01 | 0.01 | 0.01,  0.91 |
|  | Quadratic Component * Age | -0.002 | 0.003 | 447.79 | -0.72 | 0.47 | -0.01 | 0.003 | 0.52,  0.47 |
|  | Cubic Component * Age | -0.01 | 0.002 | 94854.91 | -3.64 | <0.001 | -0.01 | -0.004 | 13.24,  <0.01 |
|  | Group * Age | -0.003 | 0.002 | 450.28 | -1.81 | 0.07 | -0.01 | <0.001 | 3.30,  0.07 |
|  | Slope * Group * Age | 0.002 | 0.005 | 432.71 | 0.30 | 0.76 | -0.01 | 0.01 | 0.09,  0.76 |
|  | Quadratic Component * Group * Age | 0.003 | 0.005 | 455.75 | 0.72 | 0.47 | -0.01 | 0.01 | 0.52,  0.47 |
|  | Cubic Component * Group * Age | 0.002 | 0.003 | 94819.54 | 0.76 | 0.444 | -0.003 | 0.01 | 0.59,  0.44 |

*Table S19****: Females model output****, with ‘*’ marking Interactions. Significant β are marked with a P-Value < 0.05, to be interpreted as different from the reference level (non-autistic).*

| Sex | Term | Coef. | SE | DF | T-value | P-Value | CI 2.5% | CI 97.5% | χ^2^(1), p-value |
| --- | --- | --- | --- | --- | --- | --- | --- | --- | --- |
| Female | Intercept | 0.35 | 0.07 | 8.47 | 5.15 | 0.001 | 0.22 | 0.48 | 26.55,  <0.01 |
|  | Slope | -0.34 | 0.11 | 194.96 | -3.19 | 0.002 | -0.54 | -0.10 | 10.17,  <0.01 |
|  | Quadratic Component | 0.05 | 0.08 | 209.33 | 0.59 | 0.55 | -0.09 | 0.21 | 0.35,  0.55 |
|  | Cubic Component | -0.07 | 0.06 | 40743.82 | -1.15 | 0.25 | -0.17 | 0.03 | 1.32,  0.25 |
|  | Group | 0.06 | 0.04 | 200.57 | 1.30 | 0.19 | -0.04 | 0.14 | 1.70,  0.19 |
|  | Age | 0.004 | 0.002 | 206.54 | 2.14 | 0.03 | 0.001 | 0.01 | 4.59,  0.03 |
|  | Proportion of Missing Data | -0.001 | <0.001 | 38031.34 | -8.03 | <0.001 | -0.001 | -0.001 | 64.44,  <0.01 |
|  | Slope * Group | 0.12 | 0.14 | 191.31 | 0.86 | 0.39 | -0.19 | 0.37 | 0.74,  0.39 |
|  | Quadratic Component * Group | -0.14 | 0.10 | 203.00 | -1.38 | 0.17 | -0.39 | 0.05 | 1.90,  0.17 |
|  | Cubic Component * Group | 0.03 | 0.07 | 41568.98 | 0.44 | 0.66 | -0.10 | 0.19 | 0.19,  0.66 |
|  | Slope * Age | 0.01 | 0.01 | 194.67 | 1.72 | 0.09 | -0.004 | 0.02 | 2.95,  0.09 |
|  | Quadratic Component * Age | 0.004 | 0.004 | 210.07 | 0.96 | 0.34 | -0.005 | 0.01 | 0.92,  0.34 |
|  | Cubic Component * Age | -0.001 | 0.003 | 39652.21 | -0.33 | 0.74 | -0.01 | 0.005 | 0.11,  0.74 |
|  | Group * Age | -0.005 | 0.002 | 202.35 | -1.91 | 0.06 | -0.01 | 0.001 | 3.64,  0.06 |
|  | Slope * Group * Age | -0.01 | 0.008 | 191.61 | -0.91 | 0.36 | -0.02 | 0.01 | 0.82,  0.36 |
|  | Quadratic Component * Group * Age | 0.01 | 0.01 | 203.49 | 1.513 | 0.132 | -0.002 | 0.02 | 2.29,  0.13 |
|  | Cubic Component * Group * Age | 0.001 | 0.004 | 41170.46 | 0.21 | 0.83 | -0.01 | 0.01 | 0.04,  0.83 |

### S1.3.3 Dynamic Video

#### S1.3.3.1 Sex Differences Models Selection

For the non-autistic group, the model including sex, age, and the proportion of missing data per time bin as covariates of the 2nd degree polynomials proved the best fit. For the autistic group, the model including sex in interaction with the 2nd degree polynomials, and age and the proportion of missing data as covariates proved the best fit (Table S20 and S21).

*Table S20:* ***Non-autistic group model selection output****, comparing the base model (i.e., including polynomials of degree 3 as fixed effects and random effects), with models with additional fixed effects. ‘+’ marks adding the specified variable as a covariate; ‘*’ marks adding the interaction between the polynomial components and the specified variable. A p-value < 0.05 marks a significant comparison, i.e., better explanatory power compared to the base model.*

| Group | Model | N Parameters | AIC | BIC | Deviance | Statistic | DF | P-value |
| --- | --- | --- | --- | --- | --- | --- | --- | --- |
| Non-autistic | Base | 8 | 3798.34 | 3854.08 | 3782.34 | NA | NA | NA |
|  | + Sex | 9 | 3788.63 | 3851.34 | 3770.63 | 11.70 | 1 | 0.00 |
|  | * Sex | 11 | 3788.17 | 3864.81 | 3766.17 | 4.46 | 2 | 0.11 |
|  | + Sex + Age | 10 | 3775.61 | 3845.28 | 3755.61 | 15.03 | 1 | 0.00 |
|  | + Sex * Age | 11 | 3774.20 | 3850.84 | 3752.20 | 3.41 | 1 | 0.06 |
|  | + Sex + Age + Proportion of Missing Data | 11 | 3718.22 | 3794.87 | 3696.22 | 59.38 | 1 | <0.01 |

*Table S21:* ***Autistic group model selection output****, comparing the base model (i.e., including polynomials of degree 3 as fixed effects and random effects), with models with additional fixed effects. ‘+’ marks adding the specified variable as a covariate; ‘*’ marks adding the interaction between the polynomial components and the specified variable. A p-value < 0.05 marks a significant comparison, i.e., better explanatory power compared to the base model.*

| Group | Model | N Parameters | AIC | BIC | Deviance | Statistic | DF | P-value |
| --- | --- | --- | --- | --- | --- | --- | --- | --- |
| Autistic | Base | 8 | 9360.20 | 9419.81 | 9344.20 | NA | NA | NA |
|  | + Sex | 9 | 9357.92 | 9424.99 | 9339.92 | 4.27 | 1 | 0.04 |
|  | * Sex | 11 | 9350.21 | 9432.19 | 9328.21 | 11.71 | 2 | 0.00 |
|  | + Sex + Age | 12 | 9347.77 | 9437.19 | 9323.77 | 4.45 | 1 | 0.03 |
|  | + Sex * Age | 17 | 9354.40 | 9481.08 | 9320.40 | 3.37 | 5 | 0.64 |
|  | + Sex + Age + Proportion of Missing Data | 13 | 9153.90 | 9250.77 | 9127.90 | 195.87 | 1 | 0.00 |

#### S1.3.3.2 Sex Differences Model Output

In addition to the report of the significant coefficients in the main text, we report below the list of all coefficients and measures (Table S22 and S23).

*Table S22:* ***Non-autistic group model output****, with ‘*’ marking Interactions. Significant β are marked with a P-Value < 0.05, to be interpreted as different from the reference level (female).*

| Group | Term | Coef. | SE | DF | T-value | P-Value | CI 2.5% | CI 97.5% | χ^2^(1), p-value | |
| --- | --- | --- | --- | --- | --- | --- | --- | --- | --- | --- |
| Non-autistic | Intercept | 0.75 | 0.04 | 10.65 | 20.76 | <0.001 | 0.68 | 0.82 | 387.24,  <0.01 | |
|  | Slope | 0.25 | 0.01 | 228.32 | 24.07 | <0.001 | 0.23 | 0.27 | 583.15,  <0.01 | |
|  | Quadratic Component | -0.30 | 0.01 | 7384.25 | -34.01 | <0.001 | -0.32 | -0.28 | 1147.98,  <0.01 | |
|  | Sex | -0.06 | 0.01 | 227.71 | -4.18 | <0.001 | -0.09 | -0.03 | 13.93,  <0.01 | |
|  | Age | 0.004 | 0.001 | 228.25 | 3.624 | <0.001 | 0.002 | 0.006 | 15.40,  <0.01 | |
|  | Proportion of Missing Data | -0.33 | 0.04 | 3619.56 | -7.80 | <0.001 | -0.41 | -0.25 |  |  |

*Table S23:* ***Autistic group model output****, with ‘*’ marking Interactions. Significant β are marked with a P-Value < 0.05, to be interpreted as different from the reference level (female).*

| Group | Term | Coef. | SE | DF | T-value | P-Value | CI 2.5% | CI 97.5% | χ^2^(1), p-value |
| --- | --- | --- | --- | --- | --- | --- | --- | --- | --- |
| Autistic | Intercept | 0.70 | 0.04 | 9.19 | 16.89 | <0.001 | 0.63 | 0.78 | 285.16,  <0.01 |
|  | Slope | 0.22 | 0.02 | 370.85 | 12.09 | <0.001 | 0.18 | 0.25 | 146.10,  <0.01 |
|  | Quadratic Component | -0.29 | 0.01 | 11998.31 | -19.99 | <0.001 | -0.32 | -0.26 | 399.62,  <0.01 |
|  | Sex | -0.04 | 0.02 | 392.95 | -2.28 | 0.02 | -0.07 | -0.004 | 5.19,  0.02 |
|  | Age | 0.001 | 0.001 | 391.05 | 1.19 | 0.23 | -0.001 | 0.004 | 1.42,  0.23 |
|  | Proportion of Missing Data | -0.40 | 0.03 | 5969.12 | -14.19 | <0.001 | -0.46 | -0.34 | 201.43,  <0.01 |
|  | Slope*Sex | -0.02 | 0.02 | 374.96 | -1.09 | 0.28 | -0.06 | 0.02 | 1.18,  0.28 |
|  | Quadratic*Sex | 0.05 | 0.02 | 11994.41 | 3.12 | 0.002 | 0.02 | 0.09 | 9.72,  <0.01 |

#### S1.3.3.3 Diagnostic Groups Models Selection

For both males and females, the model including diagnosis in interaction with 2nd degree polynomials, and age and the proportion of missing data per time bin as covariates obtained the best fit (Tables S24 and S25).

*Table S24:* ***Males model selection output****, comparing the base model (i.e., including polynomials of degree 3 as fixed effects and random effects), with models with additional fixed effects. ‘+’ marks adding the specified variable as a covariate; ‘*’ marks adding the interaction between the polynomial components and the specified variable. A p-value < 0.05 marks a significant comparison, i.e., better explanatory power compared to the base model.*

| Sex | Model | N Parameters | AIC | BIC | Deviance | Statistic | DF | P-value |
| --- | --- | --- | --- | --- | --- | --- | --- | --- |
| Male | Base | 8 | 9931.98 | 9992.60 | 9915.98 | - | - | - |
|  | + Group | 9 | 9907.57 | 9975.76 | 9889.57 | 26.42 | 1 | <0.01 |
|  | * Group | 11 | 9890.59 | 9973.93 | 9868.59 | 20.97 | 2 | <0.01 |
|  | * Group + Age | 12 | 9874.98 | 9965.90 | 9850.98 | 17.61 | 1 | <0.01 |
|  | * Group * Age | 17 | 9882.30 | 10011.10 | 9848.30 | 2.68 | 5 | 0.75 |
|  | * Group + Age + Proportion of Missing Data | 13 | 9717.29 | 9815.78 | 9691.29 | 159.69 | 1 | <0.01 |

*Table S25:* ***Females model selection****, comparing the base model (i.e., including polynomials of degree 3 as fixed effects and random effects), with models with additional fixed effects. ‘+’ marks adding the specified variable as a covariate; ‘*’ marks adding the interaction between the polynomial components and the specified variable. A p-value < 0.05 marks a significant comparison, i.e., better explanatory power compared to the base model.*

| Sex | Model | N Parameters | AIC | BIC | Deviance | Statistic | DF | P-value |
| --- | --- | --- | --- | --- | --- | --- | --- | --- |
| Female | Base | 9 | 3362.91 | 3423.43 | 3344.91 | - | - | - |
|  | + Group | 11 | 3364.89 | 3438.87 | 3342.89 | 2.02 | 2 | 0.36 |
|  | * Group | 11 | 3360.75 | 3434.73 | 3338.75 | 4.14 | 0 | <0.01 |
|  | * Group + Age | 12 | 3362.64 | 3443.34 | 3338.64 | 0.12 | 1 | 0.73 |
|  | * Group * Age | 17 | 3371.10 | 3485.42 | 3337.10 | 1.54 | 5 | 0.91 |
|  | * Group + Proportion of Missing Data | 12 | 3259.51 | 3340.21 | 3235.51 | 103.25 | 1 | <0.01 |

#### S1.3.3.4 Diagnostic Groups Model Output

In addition to the report of the significant coefficients in the main text, we report below the list of all coefficients and measures (Table S26 and S27).

*Table S26:* ***Males model output****, with ‘*’ marking Interactions. Significant β are marked with a P-Value < 0.05, to be interpreted as different from the reference level (female).*

| Sex | Term | Coef. | SE | DF | T-value | P-Value | CI 2.5% | CI 97.5% | χ^2^(1), p-value | |
| --- | --- | --- | --- | --- | --- | --- | --- | --- | --- | --- |
| Male | Intercept | 0.70 | 0.04 | 8.16 | 17.57 | <0.001 | 0.62 | 0.77 | 308.79,  <0.01 | |
|  | Slope | 0.25 | 0.01 | 401.08 | 16.54 | <0.001 | 0.22 | 0.27 | 273.55,  <0.01 | |
|  | Quadratic Component | -0.29 | 0.01 | 13535.89 | -24.67 | <0.001 | -0.31 | -0.27 | 608.84,  <0.01 | |
|  | Group | -0.07 | 0.01 | 434.59 | -4.79 | <0.001 | -0.09 | -0.04 | 22.97,  <0.01 | |
|  | Age | 0.004 | 0.001 | 432.28 | 3.35 | 0.001 | 0.001 | 0.006 | 11.21,  <0.01 | |
|  | Proportion of Missing Data | -0.37 | 0.03 | 7017.03 | -12.80 | <0.001 | -0.43 | -0.31 | 163.91,  <0.01 | |
|  | Slope * Group | -0.05 | 0.02 | 413.19 | -2.69 | 0.007 | -0.09 | -0.02 | 7.23,  0.01 | |
|  | Quadratic * Group | 0.05 | 0.01 | 13554.26 | 3.61 | <0.001 | 0.03 | 0.08 | 13.01, <0.01 |  |

*Table S27****: Females model output****, with ‘*’ marking Interactions. Significant β are marked with a P-Value < 0.05, to be interpreted as different from the reference level (female).*

| Sex | Term | Coef. | SE | DF | T-value | P-Value | CI 2.5% | CI 97.5% | χ^2^(1), p-value |
| --- | --- | --- | --- | --- | --- | --- | --- | --- | --- |
| Female | Intercept | 0.82 | 0.03 | 6.20 | 25.96 | <0.001 | 0.77 | 0.89 | 673.72,  <0.01 |
|  | Slope | 0.26 | 0.02 | 335.62 | 13.63 | <0.001 | 0.22 | 0.31 | 185.73,  <0.01 |
|  | Quadratic Component | -0.33 | 0.02 | 5958.75 | -20.12 | <0.001 | -0.37 | -0.30 | 404.78,  <0.01 |
|  | Group | -0.09 | 0.02 | 188.43 | -5.03 | <0.001 | -0.13 | -0.06 | 25.30,  <0.01 |
|  | Proportion of Missing Data | -0.41 | 0.04 | 2618.75 | -10.31 | <0.001 | -0.51 | -0.33 | 106.22,  <0.01 |
|  | Slope * Group | -0.04 | 0.02 | 339.14 | -1.78 | 0.07 | -0.09 | 0.01 | 3.18,  0.07 |
|  | Quadratic * Group | 0.04 | 0.02 | 5961.43 | 1.89 | 0.06 | -0.003 | 0.08 | 3.57,  0.06 |

## S1.4 Controlling for FSIQ

After controlling for % missing data in the models above, the contribution of FSIQ was not significant anymore at the model selection step:

- Non-autistic subsample, model not including FSIQ vs. model including FSIQ: p-value of Likelihood Ratio Test = 0.06, AIC (Akaike Information Criteria) = 24508 vs. 24507
- Autistic subsample, model not including FSIQ vs. model including FSIQ: p-value of Likelihood Ratio Test = 0.50, AIC (Akaike Information Criteria) = 29808 vs. 29810
- Male subsample, model not including FSIQ vs. model including FSIQ: p-value of Likelihood Ratio Test = 0.88, AIC (Akaike Information Criteria) = 35827 vs. 35829
- Female subsample, model not including FSIQ vs. model including FSIQ: p-value of Likelihood Ratio Test = 0.16, AIC (Akaike Information Criteria) = 183392 vs. 18392

## S1.5 Dimensional Variation

Below we report the complete list of contrasts (linear comparisons between coefficients) between females and males, with the corresponding p-value indicating whether that effect differs between sexes (Table S28). Further, we report the complete output of the linear regression investigating the association between the females’ sex-diagnostic difference score and ADOS SA-CSS (Table S29).

*Table S28: Contrasts specification, with coefficient estimate of the slope (significant coefficients marked with ‘*’), and the difference to which the statistical test has been applied.*

| Variable | Component | Coef. (Male) | Coef. (Female) | Contrast (Male - Female) | SE | df | t.ratio | p.value |
| --- | --- | --- | --- | --- | --- | --- | --- | --- |
| SRS T-Score | Intercept | -0.06 (-0.12~0.01) | 0.07 (-0.03~0.17) | -0.13 | 0.06 | 1945 | -2.05 | 0.17 |
|  | Quadratic | -0.04 (-0.11~0.03) | -0.07 (-0.17~0.04) | 0.03 | 0.06 | 1945 | 0.47 | 0.96 |
| RBS-R Total Score | Intercept | 0 (-0.07~0.06) | -0.03 (-0.14~0.07) | 0.03 | 0.06 | 1995 | 0.54 | 0.95 |
|  | Quadratic | -0.07 (-0.14~-0.01) | 0.02 (-0.08~0.13) | -0.10 | 0.06 | 1995 | -1.56 | 0.40 |
| ADOS SA-CSS | Intercept | 0.02 (-0.05~0.08) | -0.18 (-0.28~-0.08) | 0.19 | 0.06 | 1975 | 3.16 | 0.01 |
|  | Quadratic | -0.11 (-0.17~-0.04) | 0.17 (0.07~0.27) | -0.28 | 0.06 | 1975 | -4.49 | <0.01 |
| ADOS RRB-CSS | Intercept | 0.02 (-0.04~0.08) | -0.22 (-0.32~-0.11) | 0.24 | 0.06 | 1975 | 3.83 | <0.01 |
|  | Quadratic | -0.08 (-0.14~-0.01) | 0.11 (0.01~0.22) | -0.19 | 0.06 | 1975 | -3.05 | 0.01 |

*Table S29: Difference score/ADOS SA-CSS multiple linear regression (significant coefficients marked with ‘*’)*

| Term | Coef. | SE | T-value | P-Value |
| --- | --- | --- | --- | --- |
| Intercept | -0.007 | 0.002 | -3.08 | 0.002* |
| Age | 0.0001 | 0.0001 | 1.08 | 0.27 |
| Stimulus: Static Social Scenes | 0.02 | 0.001 | 14.67 | <0.01* |
| Stimulus: Dynamic Video | 0.007 | 0.001 | 4.42 | <0.01* |
| ADOS SA-CSS | -0.001 | 0.0003 | -4.56 | <0.01* |
| ADOS SA-CSS*Slope | 0.00009 | 0.0003 | 0.31 | 0.75 |
| ADOS SA-CSS*Quadratic | 0.0006 | 0.0003 | 2.08 | 0.03* |
